# Evaluating interdisciplinary research: Disparate outcomes for topic and knowledge base

**DOI:** 10.1073/pnas.2409752122

**Published:** 2025-04-18

**Authors:** Sidney Xiang, Daniel M. Romero, Misha Teplitskiy

**Affiliations:** ^a^University of Michigan School of Information, Ann Arbor, MI 48109; ^b^Center for the Study of Complex Systems, University of Michigan, Ann Arbor, MI 48109; ^c^Department of Electrical Engineering and Computer Science, University of Michigan, Ann Arbor, MI 48109

**Keywords:** interdisciplinarity, publishing, peer review

## Abstract

Interdisciplinary research is essential for addressing complex global challenges, but there are concerns that scientific institutions like journals select against it. Prior work has focused largely on how interdisciplinarity relates to outcomes for published papers, but which papers get accepted for publication in the first place is unclear. Furthermore, journals may evaluate two key dimensions of interdisciplinarity,—topic and knowledge base,—differently. Topic interdisciplinarity (measured through title and abstract) may incur evaluation penalties by cutting across disciplinary evaluation standards and threatening symbolic boundaries, while knowledge-base interdisciplinarity (measured through references) may incur benefits by combining a large pool of nonredundant information. Evaluations may also depend on how well these dimensions align with each other and the intended audience. We test these arguments using data on 128,950 submissions to 62 journals across STEM disciplines, including both accepted and rejected manuscripts. We find that a 1SD increase in knowledge-base interdisciplinarity is associated with a 0.9 percentage-point higher acceptance probability, while a 1SD increase in topic interdisciplinarity corresponds to a 1.2 percentage-point lower acceptance probability. However, the penalty for high topic-interdisciplinarity diminishes when knowledge-base interdisciplinarity is also high, and when submitted to journals designated as “interdisciplinary.” These findings challenge the narrative of a uniform bias against interdisciplinary research and highlight the importance of distinguishing between its dimensions, as well as their alignment with each other and the intended audience.

## Significance

Interdisciplinary research is essential to solving the world’s most pressing problems, there are concerns that scientific institutions like journals select against it. Analyzing peer review data from 128,950 science and technology manuscripts, including those that were rejected, we found that higher knowledge-base interdisciplinarity (measured through references) was associated with higher acceptance rates, and higher topic interdisciplinarity (measured through title and abstract text) was associated with lower ones. Highly topic-interdisciplinary submissions did not fare worse if they also had high knowledge-base interdisciplinarity, and interdisciplinary journals were more favorable toward both types. This suggests the importance of distinguishing different dimensions of interdisciplinarity and their alignment with each other and the audience.

Interdisciplinary research (IDR) is research that “integrates perspectives, information, data, techniques, tools, concepts, and/or theories from two or more disciplines” (1). For decades, the importance of IDR has been touted for bridging research communities and fostering knowledge integration across boundaries (2). Policymakers and institutions have increasingly recognized its importance, creating new funding streams (3), research centers (4, 5), and departmental structures (6) to support IDR.

Despite ostensible institutional enthusiasm, many scholars still have doubts about adopting interdisciplinary research agendas, believing that there are penalties against interdisciplinarity in evaluative contexts such as hiring, promotion, and tenure (7–9), as well as peer review (10). Empirical evidence is mixed, with some existing work finding evidence that IDR is associated with evaluation penalties, while other work finds either no association or evaluation benefits. Furthermore, most existing studies are only able to observe published papers and have limited insight into effects at the journal peer review stage.

In this paper, we resolve these gaps in the literature by leveraging a unique dataset including both accepted and rejected papers. This allows us to investigate interdisciplinarity at the journal review stage directly, alleviating much of the selection bias of observing only published papers. Drawing from literature on category spanning evaluation and team diversity, we distinguish between two dimensions of interdisciplinarity: Topic and knowledge base. Topic interdisciplinarity is the interdisciplinarity of the subject matter a work addresses, which we measure via classification of title and abstract text into disciplines. It may be associated with lower evaluations because its interdisciplinarity is highly apparent, leading to difficulties in interpretation or identifying appropriate standards of evaluation. Knowledge-base interdisciplinarity is the interdisciplinarity of ideas a work uses to support its claims. We measure it via the disciplinary categories of the focal work’s references. Knowledge-base interdisciplinarity may lead to higher evaluations because the work draws from a larger pool of nonredundant information and demonstrates mastery of a wider range of literature.

We explore these arguments using administrative data from the Institute of Physics Publishing, one of the world’s largest STEM publishers. The data include metadata and review outcomes for 128,950 manuscripts submitted between 2018–2022 to 62 peer-reviewed journals encompassing a wide range of STEM disciplines, with titles such as “Neuromorphic Computing and Engineering,” “Environmental Research: Climate,” and “Physics Education.” We find that manuscripts with high knowledge-base interdisciplinarity have better evaluation outcomes on average: a 1SD higher interdisciplinarity in references is associated with a 0.7 percentage point higher probability of positive reviews and a 0.9 percentage point higher probability of acceptance. However, a 1SD higher topic interdisciplinarity is associated with a 0.4 percentage point lower probability of positive reviews and a 1.2 percentage point lower probability of acceptance.

These associations vary asymmetrically with alignment between the two types. Submissions with high topic interdisciplinarity are not penalized when their knowledge-base interdisciplinarity is also high. However, submissions with high knowledge-base interdisciplinarity are not associated with further evaluation benefits when their topic interdisciplinarity is also high. Furthermore, these associations depend on whether the journal is categorized as interdisciplinary by the publisher, with interdisciplinary journals displaying no penalty against either form of interdisciplinarity. This illustrates the efficacy of designated interdisciplinary venues in promoting IDR.

These findings have important implications for the study of IDR, as well as research evaluation, strategies, and institutions. This study includes rejected manuscripts in the study of interdisciplinarity in journal peer review to attenuate the selection issues in observing only accepted papers. Additionally, we expand the conception of interdisciplinarity to encompass multiple dimensions of a manuscript, a distinction left unmade in much scholarship on category spanning in creative works. Our findings suggest that works are better conceptualized as bundles of elements, each having its own degree of category-spanning, and that these dimensions and their alignment may be associated with different benefits and penalties. These distinctions help bring existing findings into a common framework.

For researchers, the findings suggest that IDR is not universally encouraged or discouraged by journals and reviewers—associations are only of modest magnitude and differ in direction based on interdisciplinarity type and alignment. This challenges the dominant view that IDR is widely penalized. For institutions, the result that journals designated as “interdisciplinary” give higher evaluations to both types of interdisciplinarity suggests that top–down efforts to promote category-spanning creative work can be effective. Preferences for or against interdisciplinarity are unlikely to be caused by unavoidable cognitive biases during evaluation (11) but can be strategically managed. Further research is needed to understand the extent to which the observed associations are causal, and what mechanisms drive the evaluation differences between topic/knowledge-base interdisciplinarity and mono-/interdisciplinary journals.

## Background

### Evaluation Penalties.

Following ref. 12, interdisciplinary research products can be viewed as a type of category-spanning product in a market. Audiences in a market interpret products through a shared framework of categories, and their assessments are influenced by a product’s similarity to prototypical category features. (13). Many studies have shown that category-spanning entities—those that blend features of multiple categories—are evaluated less favorably than those that specialize in a single category. This effect has been found in domains as diverse as actors (14), feature films (13), eBay (13), peer-to-peer lending websites (15), and wines (16).

There are two commonly cited arguments for why these evaluation penalties arise. First, the effect may be on the “audience-side,” where audiences discount category-spanning products because of perceptions of lower quality. Audiences may use the heuristic that multiple category membership results in fewer resources being devoted to each category, leading to a lower quality product (13). Additionally, if audiences closely identify with a category (e.g. a disciplinary journal editor evaluating manuscripts), there may be gatekeeping: attempts to defend the distinctiveness of their category against outsiders (17, 18). The cognitive challenges of processing a category-spanning entity may also lead to difficulties in evaluation and unconscious biases (19, 20). Second, the penalty may occur on the “producer-side,” where the resource splitting inherent in category-spanning actually lowers product quality. This is distinct from the perception of lower quality, as shown in refs. 15 and 16.

In the context of IDR evaluation, single-category products are monodisciplinary research works, and multicategory products are interdisciplinary research works. Potential audience-side penalties include evaluators inferring a lower level of expertise behind interdisciplinary offerings, gatekeeping their discipline against perceived outsiders (17), and being unsure of what set of standards to apply to interdisciplinary work (as different fields hold different values and norms) (21). Potential producer-side penalties include the increased cognitive and collaborative overhead necessary to integrate knowledge across disciplines and the resultant dip in productivity (22). Thus, the category-spanning literature suggests that IDR may incur evaluation penalties because of audience perceptions of lower quality, along with potentially actual lower quality resulting from the difficulty of doing interdisciplinary work.

### Evaluation Benefits.

Category-spanning may also lead to benefits. Studies on team diversity have consistently highlighted the importance of educational background and expertise heterogeneity to team creativity and innovation (23). This relationship appears to be moderated by factors such as participatory leadership and the extent of advice networks within and without the group (24, 25) as cited in ref. 23). The importance of participatory engagement and robust communication networks supports the claim that diverse teams produce greater innovation through the mechanism of knowledge integration: the ability to draw upon wider pools of nonredundant information to solve problems. This idea is supported by research at the organizational level as well: slow individual convergence to organizational knowledge helps maintain the diversity of information needed to improve organizational knowledge over time (26).

These conclusions have significant implications for IDR, where team innovation and collective knowledge-building are the modus operandi. The diversity of expertise represented by an interdisciplinary collaboration directly translates into diversity of information, which contributes to higher levels of collective knowledge. Accordingly, interdisciplinary research can be of greater quality in the long run than monodisciplinary research produced using more homogeneous information. This quality premium should merit higher evaluations.

### Evaluation and Alignment.

The category-spanning market framework does not account for the potential role of alignment: alignment among features of the research product, as well as alignment between product and audience.

To conceptualize the alignment of research product features, we can look to the organizational concept of congruence, or the alignment between “needs, demands, goals, objectives, and/or structures” of various components within an organization (27). The importance of congruence (particularly between marketing and operations) to organizational effectiveness has been reviewed in ref. 28. They theorize that a marketing campaign’s effectiveness relies on the alignment between the campaign and operations, or the ability of organizational operations to fulfill the campaign’s promises. Likewise, a research product’s topic, as “marketed” through its title and abstract, can only be compelling if supported by an appropriate knowledge base. The interdisciplinarity of a topic should be matched by the interdisciplinarity of the information used to make claims about it.

Beyond alignment within research product components, alignment between research product and anticipated audience is also important. This relationship can be viewed through the lens of strategic framing: the “use of rhetorical devices in communication to mobilize support and minimize resistance” (29). Interdisciplinary researchers are especially cognizant of the importance of strategic framing when presenting their work for disciplinary audiences (22). Since different disciplines adhere to different norms, standards, and values, savvy authors employ strategic framing to present their research product in a way that aligns with the expectations of their target audience, thereby enhancing perceived legitimacy and relevance within disciplinary communities (30). Strategic framing may thus work to mitigate audience-side penalties by increasing the perceived degree of membership within one category while decreasing membership in others. Although strategic framing is often discussed within the management and organization science literature for its effectiveness in market positioning (30–32), its relevance extends to research evaluation as well, especially when considering the boundary-spanning case of IDR. Thus, when considering the evaluation of IDR, we expect that alignment in interdisciplinarity between the product and the audience should lead to better evaluation outcomes.

### Prior Research on Peer Evaluation of IDR.

A number of prior works investigate the peer evaluation of IDR through direct outcomes like accreditation, grants, and publication; or downstream outcomes like citation impact. However, there are two major gaps in the literature this study helps fill. First, most studies treat interdisciplinarity as a one-dimensional feature, using a single basis of measurement such as author-provided labels or disciplines of referenced journals. The variation in empirical findings suggests that basis of measurement makes a difference—thus, one contribution is to measure two distinct kinds of interdisciplinarity that are often invoked in the empirical literature as standalone measures. Second, the only works that have studied IDR in the setting of journal peer review have only been able to do so indirectly through outcome variables such as time spent in review, and most suffer from selection bias due to the unobservability of unpublished papers. This paper addresses this gap by using a unique administrative dataset provided by a large STEM publisher that includes decisions for both accepted and rejected manuscripts.

#### Mixed findings and measurements.

Prior findings are mixed regarding whether interdisciplinary research helps or hurts scientists’ careers. IDR has been found to negatively affect productivity (12), professional advancement (17, 33), and grant acquisition (34), particularly when evaluation is structured around disciplinary panels (35). However, moderately interdisciplinary papers have greater citation impact (36), especially in the long term (37), although they experience higher variance (12, 38). Interdisciplinary team composition has been found to lead to higher impact papers (39–41). Dai et al.’s study measuring more than one dimension of interdisciplinarity observes inconsistent associations within the same set of papers, observing that poor semantic fit with established fields leads to lower chances of publication in a top journal, while referencing outside of one’s field leads to higher chances and higher citation impact once published (42).

We argue that these mixed findings are partially due to how the evaluation of IDR differs along different dimensions of interdisciplinarity. The empirical studies mentioned above use very different features as their basis of measurement. For instance, refs. 34 and 35 use author-reported disciplinary focus areas, refs. 17, 40, and 41 use the disciplines of authors’ prior publications, and refs. 12, 36, 37, and 42 use the disciplines of a work’s references, while ref. 42 uses semantic features of the text. Each of these quantities captures different dimensions of a project, and interdisciplinarity within different dimensions may lead to different evaluation outcomes.

Existing literature falls into two broad categories: those that measure author-reported disciplines and those that measure the disciplines of references or prior work. Author-reported disciplines are a declaration of categories authors want their work to compete in. When evaluation is structured around categories, interdisciplinary products incur audience-side penalties from lower perceived quality, higher cognitive load, unclear evaluation standards, and categorical gatekeeping. Thus, Bromham et al. and Seeber et al. (34, 35) both find disciplinary evaluation penalties against IDR when interdisciplinarity is measured in this way.

On the other hand, prior publications and references represent the pool of knowledge and expertise from which a project draws. Greater diversity in these should lead to greater collective knowledge and innovation. Measuring interdisciplinarity via authors’ prior publications, AlShebli et al. and Zheng et al. (40, 41) both find that this type of interdisciplinarity is associated with higher citation impact. Measuring via references, (12, 36, 37, 42) also find better evaluation outcomes for IDR.

The differing bases of measurement in the literature motivate the use of two distinct measures of interdisciplinarity: one that is based on the disciplinary framing of manuscript content, and one that is based on the disciplinary composition of manuscript references.

#### Limited observability of journal peer review outcomes.

Existing evidence on the success of interdisciplinary papers is limited by the unobservability of the peer review process. Of eight studies investigating IDR at the paper level, five only use data on published papers and cannot study peer review directly (36–38, 43, 44). Observing only published papers introduces a degree of selection bias—the observed citation impact effects could partially arise from a penalty against interdisciplinarity in peer review, allowing only the highest-quality interdisciplinary papers to reach publication.

Three other studies observe indirect signals of peer review outcomes: time under review (12), peer review scores in the UK’s Research Excellence Framework program (45), and preprints’ appearance in top journals (42). However, both time under review and REF peer review scores were for already-published papers, encountering the same selection issues (papers reviewed by REF were also limited to researchers’ best 1 to 5 works, introducing further selection). Observing preprints’ eventual appearance in top journals circumvents this to some extent, but without being able to observe review decisions directly, it is unclear how outside factors influence this outcome, such as authors’ strategic assessments about what journals to submit to.

The only two papers that directly observe peer review outcomes both pertain to research proposals. Bromham et al. find that interdisciplinarity is associated with lower funding success (34), while Seeber et al. find that this penalty is attenuated in the absence of disciplinary review panels (35). While these findings could generalize to the context of journal peer review, there is reason to believe that review mechanisms and the types of research rewarded differ between ex ante peer review (of proposals) and ex post peer review (of completed manuscripts) (46).

Prior research provides preliminary evidence that interdisciplinary manuscripts fare slightly worse in peer review than monodisciplinary counterparts. However, because of differences between ex ante and ex post review, as well as limitations of existing evidence on IDR in ex post review, more empirical evidence is needed. This paper leverages peer review data for 128,950 manuscripts to provide direct observational evidence of ex post peer review decisions at scale.

### Topic and Knowledge-Base Interdisciplinarity.

Existing literature measures interdisciplinarity in several different ways, leading to evidence of both penalties and benefits. To distill these varied insights, we focus on two key dimensions. First, works can be interdisciplinary in the topics addressed—the specific questions or themes they explore. Second, works can be interdisciplinary in the knowledge base they draw upon—the information they rely on to support arguments and conclusions. The need for this distinction is apparent in light of different measurement approaches yielding different findings. We argue that these two dimensions and their alignment have distinct interactions with research evaluation processes.

First, knowledge-base interdisciplinarity is more clearly associated with knowledge integration benefits since topic interdisciplinarity alone does not guarantee the use of diverse expertise. Second, topic interdisciplinarity is more clearly associated with category-spanning penalties since its category spanning is more perceptually obvious. Third, knowledge-base interdisciplinarity (particularly via references) serves a rhetorical function by demonstrating alignment with the intended audience’s intellectual milieu. Finally, a match between topic and knowledge-base interdisciplinarity should confer evaluation benefits through the display of congruence.

To explore this distinction between topic and knowledge-base interdisciplinarity, we measure the two for a large dataset of manuscripts submitted to the Institute of Physics Publishing between 2018–2022. Topic interdisciplinarity is measured via the OpenAlex concept tags on manuscripts’ associated OpenAlex records, and knowledge-base interdisciplinarity is measured via the concept tags on manuscripts’ references. By measuring them together, we can directly compare their individual and joint relationship with evaluation outcomes. Our research questions are

- RQ 1: Is there a positive or negative association between manuscripts’ topic interdisciplinarity and evaluation outcomes?
- RQ 2: Is there a positive or negative association between manuscripts’ knowledge-base interdisciplinarity and evaluation outcomes?
- RQ 3a: How does alignment between topic interdisciplinarity and knowledge-base interdisciplinarity relate to evaluation outcomes?
- RQ 3b: How does alignment between each type of interdisciplinarity and journal audience relate to evaluation outcomes?

With our dataset of submitted manuscripts, reviews, and publication outcomes, we are able to estimate these relationships in a uniquely direct way.

## Results

### Descriptive Statistics.

The distribution of each interdisciplinarity measure is shown in *SI Appendix*, Figs. S1 and S2. Descriptive statistics for the interdisciplinarity measures and covariates are shown in Table 1. Notably, topic interdisciplinarity and knowledge-base interdisciplinarity are moderately correlated, with a correlation coefficient of 0.56. This suggests careful interpretation of coefficients when regressing both variables together (i.e. keeping in mind that the coefficient for one is conditional on the other being held fixed), and examination of interaction effects.

**Table 1.** Descriptive statistics and correlations

	Mean	SD	Topic ID	Knowledge-base ID	Year (20XX)	Team size	Max author citations	Max author publications	Number of references
Topic ID	0.315	0.215	1.00	0.56	0.02	0.02	−0.01	−0.01	−0.00
Knowledge-base ID	0.474	0.166	0.56	1.00	0.07	0.07	−0.01	−0.01	0.03
Year (20XX)	19.6	1.28	0.02	0.07	1.00	0.03	0.06	0.00	0.09
Team size	4.72	3.16	0.02	0.07	0.03	1.00	0.23	0.22	0.11
Max author citations	28,039	72,218	−0.01	−0.01	0.06	0.23	1.00	0.96	0.01
Max author publications	2,047	5,425	−0.01	−0.01	0.00	0.22	0.96	1.00	0.00
Number of references	39.4	29.2	−0.00	0.03	0.09	0.11	0.01	0.00	1.00

Review positivity statistics are at the individual review level.

### Model Results.

**Fig. 1.**
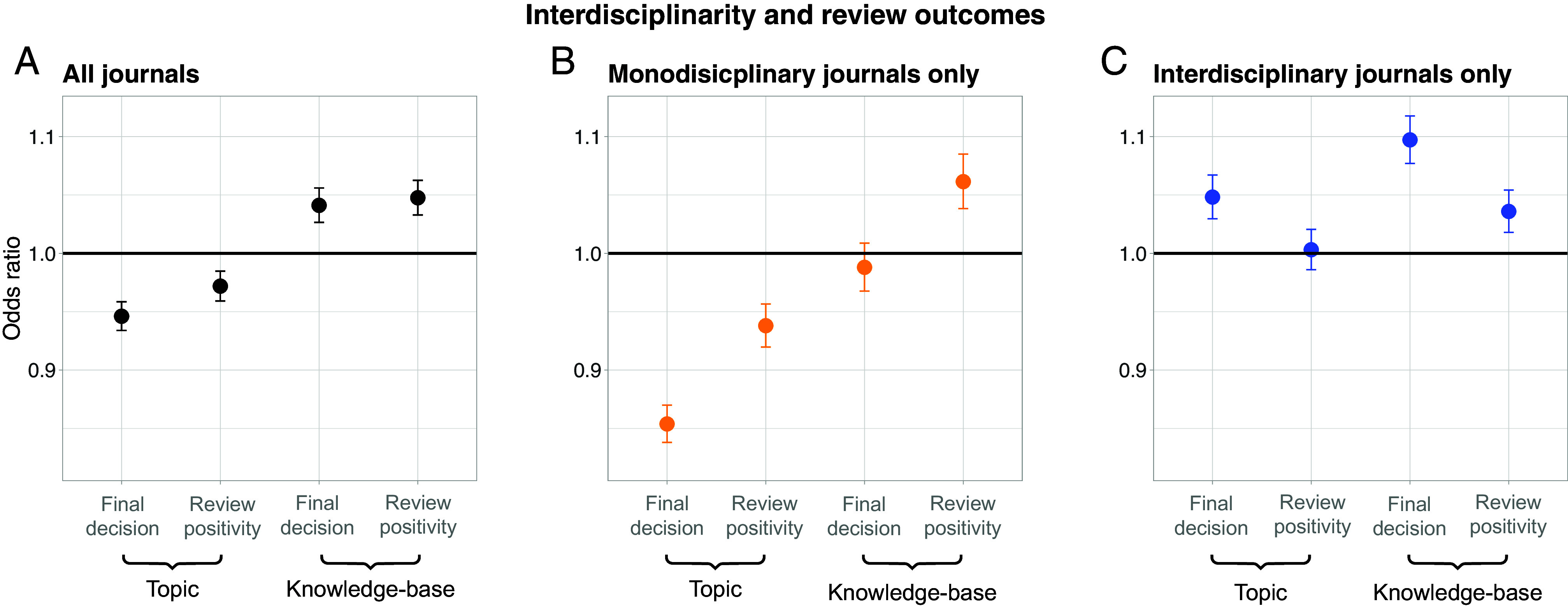
Panels depict models 3 and 4, which include covariates but not interactions. (*A*) Odds ratios and 95% CIs for all journals. (*B*) Odds ratios and 95% CIs for monodisciplinary journals only. (*C*) Odds ratios and 95% CIs for interdisciplinarity journals only.

Logistic regression results for final decision are shown in Table 2, and results for reviewer positivity are shown in Table 3. Models 1 and 2 exclude covariates and interactions, models 3, 4, and 5 add covariates, and model 6 adds the interaction. Coefficients for journal, manuscript type, and year indicator variables are omitted for readability. Odds ratios and 95% CIs for models 3 and 4 (with covariates but without interactions) are visualized in Fig. 1*A*.

**Table 2.** Logistic regression, final decision

	Dependent variable: Final decision					
	(1)	(2)	(3)	(4)	(5)	(6)
Constant	0.142∗∗∗	0.142∗∗∗	−2.642∗∗∗	−2.653∗∗∗	−2.620∗∗∗	−2.627∗∗∗
	(0.006)	(0.006)	(0.071)	(0.071)	(0.071)	(0.071)
Topic ID	−0.040∗∗∗		−0.055∗∗∗		−0.094∗∗∗	−0.096∗∗∗
	(0.006)		(0.007)		(0.008)	(0.008)
Knowledge-base ID		0.020∗∗∗		0.040∗∗∗	0.089∗∗∗	0.102∗∗∗
		(0.006)		(0.007)	(0.008)	(0.008)
Topic ID:Knowledge-base ID						0.042∗∗∗
						(0.006)

Covariates?	No	No	Yes	Yes	Yes	Yes
Observations	128,950	128,950	128,939	128,939	128,939	128,939
Log likelihood	−89,031.640	−89,050.740	−79,621.140	−79,640.350	−79,562.470	−79,541.160
Akaike Inf. Crit.	178,067.300	178,105.500	159,472.300	159,510.700	159,356.900	159,316.300

*Note:* ∗∗∗*P* < 0.01.

**Table 3.** Logistic regression, review positivity

	Dependent variable: Review positivity					
	(1)	(2)	(3)	(4)	(5)	(6)
Constant	1.391∗∗∗	1.392∗∗∗	0.613∗∗∗	0.609∗∗∗	0.633∗∗∗	0.632∗∗∗
	(0.006)	(0.006)	(0.070)	(0.070)	(0.070)	(0.070)
Topic ID	−0.012∗∗		−0.029∗∗∗		−0.060∗∗∗	−0.061∗∗∗
	(0.006)		(0.007)		(0.008)	(0.008)
Knowledge-base ID		0.049∗∗∗		0.047∗∗∗	0.076∗∗∗	0.081∗∗∗
		(0.006)		(0.007)	(0.008)	(0.008)
Topic ID:Knowledge-base ID						0.020∗∗∗
						(0.006)

Covariates?	No	No	Yes	Yes	Yes	Yes
Observations	197,119	197,119	197,090	197,090	197,090	197,090
Log likelihood	−98,435.400	−98,399.480	−95,603.910	−95,592.230	−95,560.340	−95,555.220
Akaike Inf. Crit.	196,874.800	196,803.000	191,437.800	191,414.500	191,352.700	191,344.400

*Note:* ∗∗*P* < 0.05; and ∗∗∗ *P* < 0.01.

Across all models and outcome variables, topic interdisciplinarity has a significant negative association with evaluation outcomes, whereas knowledge-base interdisciplinarity has a significant positive association. The average marginal effect of a 1SD increase in topic interdisciplinarity is a 1.2 percentage-point decrease in the probability of acceptance and a 0.4 percentage-point decrease in the probability that a review recommends acceptance. The average marginal effect of a 1SD increase in knowledge-base interdisciplinarity is a 0.9 percentage-point increase in the probability of acceptance and a 0.7 percentage-point increase in the probability that a review recommends acceptance. Given the widespread belief in IDR’s evaluation penalties and scholarly benefits, the small magnitude of these results may be surprising.

These results hold when interdisciplinarity is measured using level 1 OpenAlex concept tags (*SI Appendix*, section 7), for the corresponding linear probability model (*SI Appendix*, section 8) and using raw numeric control variables (*SI Appendix*, section 9).

We also observe significant positive interaction effects between topic and knowledge-base interdisciplinarity for both outcome variables. These interactions are on the order of the main effect sizes, with coefficients of 0.042 and 0.020 for the final decision and reviewer positivity models, respectively. While further research is needed to establish causality, this association suggests that topic and knowledge-base interdisciplinarity are beneficial when aligned: More interdisciplinary references alleviate some of the penalty against interdisciplinary topics, and more interdisciplinary topics magnify the benefits of interdisciplinary references.

**Fig. 2.**
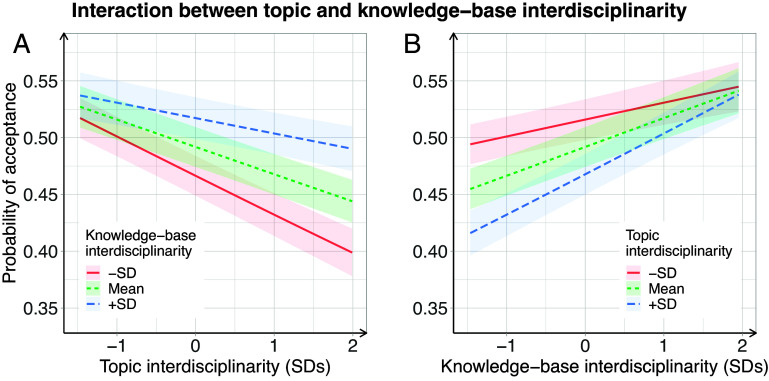
Interaction plots depicting conditional predictions of acceptance probability for modal values of covariates, based on model 6 (covariates and interactions). Shaded regions are 95% CIs. (*A*) Relationship between topic interdisciplinarity and acceptance probability for three levels of knowledge-base interdisciplinarity. (*B*) Relationship between knowledge-base interdisciplinarity and acceptance probability for three levels of topic interdisciplinarity.

Fig. 2 shows the positive interaction between topic and knowledge-base interdisciplinarity. Fig. 2*A* shows that higher knowledge-base interdisciplinarity attenuates the negative relationship between topic interdisciplinarity and acceptance. Interdisciplinary manuscripts have worse peer review outcomes when they do not support their higher topic interdisciplinarity with high knowledge-base interdisciplinarity. Fig. 2*B* shows that manuscripts with higher topic interdisciplinarity are evaluated lower at all levels of knowledge-base interdisciplinarity. In summary, interdisciplinary references are evaluated positively even when the topic is not broad, and work on broad topics is not evaluated negatively unless it fails to cite widely.

### Results Across Mono- and Interdisciplinary Journals.

We disaggregated our results by journal interdisciplinarity according to the publisher’s 2022 product catalog. The catalog categorizes journals under seven subject areas: “Physics,” “Astronomy and astrophysics,” “Materials,” “Biosciences,” “Mathematics,” “Environmental sciences,” and “Interdisciplinary.” We aggregate journals categorized as “Interdisciplinary” or under multiple subject areas into one group (“Interdisciplinary”), and all others into another group (“Monodisciplinary”). Show model 3 and 4 odds ratios coefficients (covariates, no interactions) for these groups. The corresponding regression tables are given in *SI Appendix*, section 6.

Journals categorized by the publisher as interdisciplinary or under multiple subject categories have positive associations between both types of interdisciplinarity and evaluation outcomes. On the other hand, monodisciplinary journals have negative associations between interdisciplinarity and evaluation outcomes, with the exception of knowledge-base interdisciplinarity and review positivity. These results point to the importance of alignment between the interdisciplinarities of the manuscript, gatekeepers, and intended audience.

## Discussion

This study investigates the evaluation of interdisciplinary research in the high-stakes setting of scientific publishing. We conceptualize scientific papers as bundles of several elements—in particular their topic and the knowledge base— with each having its own level of interdisciplinarity and distinct association with evaluations. We apply this framework to large-scale administrative peer review data from one of the world’s largest STEM publishing companies.

Analyzing prepublication evaluations alleviates the selection bias of studies that use only published papers. In particular, if there is selection for or against IDR at the prepublication step, the corpus of published papers may have a disproportionate amount of low or high-quality interdisciplinary papers (47), likely affecting the citation patterns observed by other studies.

Our results show that knowledge-base interdisciplinarity is associated with better evaluation outcomes, while topic interdisciplinarity is associated with worse evaluation outcomes. The associations were robust to different outcome variables, the inclusion or exclusion of relevant covariates, and measuring interdisciplinarity at the level of major fields or subfields, suggesting that the type of interdisciplinarity measured strongly determines the relationship observed.

These associations varied across journal type, with journals categorized by the publisher as interdisciplinary giving higher evaluations to both types of interdisciplinarity, suggesting that the lower evaluations associated with topic interdisciplinarity are alleviated in contexts that seek interdisciplinary work explicitly. Additionally, we find evidence for the importance of alignment between topic and knowledge-base interdisciplinarity within the manuscript: the negative association of high topic interdisciplinarity with evaluations is offset when it is matched with a high knowledge-base interdisciplinarity. One interpretation is that broader topics require broader conceptual building blocks to be effective. Another is that papers with broad topics are assigned reviewers from disparate disciplines, who expect to see their own disciplines represented in the works referenced.

### Addressing Limitations.

#### Disciplinary scope of study population.

Institute of Physics Publishing (IOPP) publishes primarily in the physical sciences, so combinations of very distant fields (such as those bridging the physical and social sciences) are scarce. However, it still contains clearly interdisciplinary journals such as *Environmental Research: Health*, *Physics Education*, and *Neuromorphic Computing and Engineering*. The publisher classifies 10 of the 62 journals included in our dataset as interdisciplinary, and of the remainder, 18 are categorized under multiple disciplinary categories. *SI Appendix*, section 1 shows the number of journals in our dataset classified under each category. Additionally, the distributions of interdisciplinarity measures span nearly the entire range from 0 to 1 (*SI Appendix*, section 1). Thus, our results are generalizable to interdisciplinarity adjacent to the natural sciences, though whether they generalize to other sciences is unclear.

#### Remaining selection bias.

Although the administrative data include final decisions and review recommendations for both accepted and rejected manuscripts, they do not include the submissions’ full text or references. We therefore rely on matching to OpenAlex data for our interdisciplinarity measures, which results in differences between our analytic sample and the full set of submissions (*Analytic Sample*). The missing data may induce a selection bias. For example, if high topic-interdisciplinary papers are more likely to get evaluated poorly, rejected, and not indexed by OpenAlex, then many such papers will be unobserved in our analysis, leading to an underestimate of the association between topic-interdisciplinarity and poor evaluations. Our conclusions should generalize best to manuscripts of sufficiently high quality to be accepted somewhere, or complete enough to be posted on a preprint server. Further research with even more data access is necessary to address the potential selection bias.

Despite these limitations, our study still represents a meaningful improvement in overcoming the selection biases of prior work, as we are able to observe a large sample of rejected manuscripts with direct peer review outcomes.

#### Manuscript quality.

Manuscript quality is clearly an important consideration in publishing, and associations between interdisciplinarity and quality would affect the relationship between interdisciplinarity and evaluation. A key limitation of our analysis (and most other studies of scientific evaluation) is the imperfect ability to account for variation in manuscript quality. Conventional measures of quality (e.g. publishing journal or citation impact) are generally available only after publication, so controlling for them may create posttreatment bias (48). Even under ideal circumstances, it is difficult to imagine the perfect experiment that could assign interdisciplinarity to manuscripts at random without affecting their other characteristics.

Instead, we follow other studies in focusing on manuscript authors, data about whom are typically available before publishing (49, 50). Specifically, we control for authors’ publication volume and citations as a proxy for manuscript quality and knowledge of the publishing process, reasoning that authors who have accumulated more publications and citations prior to manuscript submission are better able to publish again (51). All of the main results are consistent after controlling for author experience in this way.

#### Overlap with novelty.

Interdisciplinarity can be highly related to novelty, depending on how the two concepts are operationalized. Insofar as disciplines are just clusters of frequently associated ideas, unusual combinations of ideas are likely to span disciplines, and discipline-spanning combinations are likely to be unusual. In fact, Fontana et al. (52) found that Uzzi et al.’s widely used novelty measure (53) is highly correlated (R = 0.887) with Porter et al.’s interdisciplinarity measure (54), as both measures rely on pairs of concepts represented in the paper’s references. Consequently, we expect results from interdisciplinarity measures based on references, such as our knowledge-base interdisciplinarity, to correspond to those using Uzzi et al’s novelty measure. Recent research by Teplitskiy et al. supports this expectation, as it finds that higher novelty is associated with better journal evaluations (11).

### Conclusion and Implications.

The literature on the evaluation of interdisciplinary scientific research and other types of creative work typically conceptualizes them as having category-spanning that is one-dimensional, does not account for characteristics of the evaluators, and is limited by the nonobservability of rejected work. The simplicity of this conceptualization is certainly attractive, and perhaps even justifiable when several dimensions of interdisciplinarity are aligned and audiences homogeneous. However, studies operationalize interdisciplinarity in inconsistent ways and yield inconsistent conclusions regarding whether audiences evaluate interdisciplinarity more or less positively. Additionally, different dimensions of interdisciplinarity may not align, and the interdisciplinarity of the evaluators may also vary and affect evaluations.

We argued that it is important to distinguish between different dimensions of interdisciplinarity when studying IDR evaluation. Previous works have also argued for dimensions of interdisciplinarity, i.e. variety, balance, and disparity [the three components of Porter et al.’s (54)], but applied all of them to one element of a paper, such as its references, e.g., refs. 36 and 55. Yet a paper’s references (knowledge base) are conceptually distinct from its other elements, such as topic. Using rich and large-scale peer review data from the Institute of Physics Publishing, we showed that the distinction to be an empirically significant one, as these dimensions have consistently opposite associations with evaluation outcomes.

The distinction between topic, knowledge base, and audience interdisciplinarity is also theoretically significant because each type may trigger different organizational or cognitive processes, leading to different outcomes. Monodisciplinary audiences (reviewers, editors) apply their discipline’s criteria, perhaps rigidly. Submissions addressing interdisciplinary topics may be less likely to meet that discipline’s criteria, and any merits that stem from or illuminate topics outside the discipline may not compensate because they are undervalued or unrecognized. In contrast, an interdisciplinary audience should be more likely to identify the paper’s conceptually dispersed merits. Another possibility is that even monodisciplinary audiences appreciate papers addressing interdisciplinary topics more fully when they know they are reviewing for an interdisciplinary outlet.

Unlike topic interdisciplinarity, knowledge-base interdisciplinarity is viewed more positively by monodisciplinary audiences. One plausible explanation is that the average reference (on which knowledge-base interdisciplinarity is based) is simply less consequential and more “fungible” than a paper’s broad topic (56): it can be easily added or removed to signal engagement with specific intellectual traditions. An interdisciplinary knowledge base may also mean that more diverse tools and perspectives were used to answer a disciplinary question.

Finally, the sharp reduction in penalties against topic interdisciplinarity in the presence of high knowledge-base interdisciplinarity suggests the view that the dimension of topic may be linked to the research question, while the dimension of knowledge base may be linked to the resources used to answer the question—an ambitious interdisciplinary research question suffers unless backed by an equally ambitious and interdisciplinary approach to answering that question.

Our findings suggest several practical implications, although further research is needed to better assess the extent to which the observed associations are causal. First, manuscripts are better received by monodisciplinary journals if their topic matter adheres to a single discipline or a set of closely related disciplines. Second, manuscripts benefit from the synthesis of interdisciplinary literature in their references, regardless of whether the receiving journal is interdisciplinary. These patterns suggest that ensuring that one’s topic stays within-discipline but citing widely is strategic when presenting work to monodisciplinary audiences. Third, the finding that lower evaluations for interdisciplinary topics disappear for explicitly interdisciplinary journals is a sign that creating settings specifically for interdisciplinary research can be effective. Finally, and perhaps most importantly, the magnitude of the associations we found was surprisingly small given the prevalent impression that interdisciplinarity is penalized in evaluations. Consequently, the penalties for pursuing interdisciplinary research, at least in the context of publishing, should not be a deterrent to pursuing such work.

## Materials and Methods

### Data.

We use administrative data from the IOPP, a major STEM publishing company based in the United Kingdom. The research was approved by the University of Michigan Institutional Review Board, protocol HUM00194927. The full data comprise metadata on 224,259 manuscripts submitted to 62 physical sciences journals from 2018–2022, including their peer reviewer recommendations and final decisions. The 62 journals fall into 7 publisher-defined disciplinary categories: Physics, Materials, Biosciences, Environmental Sciences, Astronomy and Astrophysics, Mathematics, and Interdisciplinary (57). Of these journals, IOPP classifies 10 as “interdisciplinary,” and of the remainder, 18 are categorized under multiple disciplines. Examples of journals in our data categorized as interdisciplinary include *Neuromorphic Computing and Engineering* and *Physics Education*, and examples of journals categorized under multiple disciplines are *Biofabrication* (categorized under Materials and Biosciences) and *Inverse Problems* (categorized under Mathematics and Environmental sciences). Although our data are provided by a physical sciences publisher, there is a high degree of interdisciplinarity present.

We supplement IOPP data with data from OpenAlex [an open-access database of academic publications, authors, venues, institutions, and concepts (58)] and Crossref. Data matching and cleaning is described in *SI Appendix*, section 2.

### Measuring Interdisciplinarity.

Several measures have been proposed to quantify interdisciplinarity (59), with researchers increasingly converging on the *integration index* (12, 36, 60–62). The integration index captures three key elements of diversity: variety, balance, and disparity (54, 63). Variety is the number of categories represented, balance is the evenness of distribution among the categories, and disparity is how different the categories are from each other.

Integration captures these three aspects of diversity for a collection of disciplinary objects, such as a paper’s references. It is calculated as follows:

[1] $$I=1-\sum_{\mathrm{ij}}s_{\mathrm{ij}}\cdot p_{i}\cdot p_{j},$$

where i and j index disciplinary categories, $s_{\mathrm{ij}}$ is a measure of similarity between categories i and j, and $p_{i}$ and $p_{j}$ are the proportions of the collection made up of entities in i and j.

In this work, our collections consist of OpenAlex concept tags (64). The concept tags are a set of 65k Wikidata concepts ordered in hierarchical levels from 0 to 5, with 0 being the most general (e.g. Physics, Chemistry, Biology) and 5 being the most specific (e.g. Magnetic resonance force microscopy). There are 19 level 0 concept tags corresponding to high-level fields such as biology, political science, and engineering. 99.1% of works on OpenAlex are tagged with at least one level 0 concept, and each work is tagged with 7 to 8 concepts on average across all levels. Concept tags are assigned a score between 0 and 1 reflecting their relevance to the work.

Tags are assigned by a neural network trained on Microsoft Academic Graph (MAG) concept tags, and assignments are based on the work’s title, document type (e.g. journal article, book), journal title, and abstract. The MAG concept tags were generated by natural language processing models and validated by human surveys (65). OpenAlex’s validation shows that their tagging classifier has an F1 score of 0.751 for level 0 concept tags, and that it achieves the best performance on journal and repository papers, which includes our population of interest (64). Additionally, their analysis of incorrect tagging behavior reveals that many “false positive” tags (i.e. those that were not assigned by MAG) are still relevant, leading to underestimation of the model’s true precision.

To further assess model performance in our setting, we manually validated concept tags on a random sample of 100 submitted manuscripts found on OpenAlex by reading the titles and abstracts. The sample had a total of 228 level 0 tags with nonzero scores (tags with scores equal to zero do not affect the interdisciplinarity metric). Table 4 shows the breakdown of tag validity. Tags were labeled by one of the authors (S.X.) as “Correct” if they were relevant to the paper, “Questionable” if they were not relevant but still broadly related, and “Incorrect” if they were completely unrelated to the paper. 197 (86.4%) of the nonzero tags were correct. Of the 17 nonzero incorrect tags, 8 of them were “Computer Science,” 6 were “Geology,” 6 were other STEM fields, and only 2 were very far afield (“Psychology” and “Political Science”). There did not seem to be any systematic trend by discipline or level of interdisciplinarity in the types of manuscripts that were mistagged; however, some of the mistagged papers included polysemous words that may have been associated with the tags assigned. Examples include “parameter,” “kernel,” and “error” (computer science), and “extractive activities,” “structural damage,” and “shear” (geology). This validation exercise suggests that level 0 OpenAlex concept tags reflect the disciplinary content of papers in our sample reasonably well.

**Table 4.** Percentage of nonzero level 0 concept tags on a random sample of 100 IOPP manuscripts that were correct, questionable, or incorrect

	% Concept tags
Correct	86.4
Questionable	3.9
Incorrect	9.6

We calculate interdisciplinarity scores using level 0 concept tags. Regression results using level 1 concept tags (roughly corresponding to subfield) are qualitatively consistent with our main findings and are provided in *SI Appendix*, section 7).

Returning to the integration measure (Eq. **1**), for each concept i in the set of concepts C for a set of papers R, $p_{i}$ is given by

[2] $$p_{i}=\frac{\sum_{r\in R}\;\text{score}\left(i,r\right)}{\sum_{i\in C}\sum_{r\in R}\;\text{score}\left(i,r\right)},$$

where score(i,r) gives the score associated with concept i on paper r. We treat the set of papers as a bag of concepts, where $p_{i}$ is the weighted proportion of concepts in category i.

Similarity between concept tags ($s_{\mathrm{ij}}$) is calculated using concept co-occurrence in a random sample of OpenAlex journal articles for each year of our analysis. Concept tags that frequently co-occur will likely be similar. For example, papers are often tagged with Computer Science and Engineering, suggesting that these fields are more conceptually similar than, say, Computer Science and Philosophy, which are almost never cotagged. From the sample of articles, we construct a vector for each concept tag using the concept tag scores. The length of each vector is the number of articles in the sample, and element i in the vector is the score associated with the concept on article i in the sample. We calculate the similarity between concepts as the cosine similarity between these concept vectors.

We distinguish between two types of interdisciplinarity—topic and knowledge base—by using Eq. **1** on different sets of concept tags associated with the paper. Topic interdisciplinarity is based on the concept tags of the manuscript’s associated OpenAlex record (inferred from the title and abstract). Knowledge-base interdisciplinarity is based on the concept tags of the manuscript’s references. References form the conceptual foundation of a piece of research and serve as a rhetorical tool, as they demonstrate membership in a particular research community (66). Thus, knowledge-base interdisciplinarity represents the interdisciplinarity of both the conceptual and rhetorical support behind a manuscript.

See *SI Appendix*, section 4 for example interdisciplinarity calculations for these measures.

### Analytic Sample.

We exclude manuscript types for which IOPP’s standard peer review process does not apply (such as invited articles), reducing our population of manuscripts from 224,259 to 221,702. Second, we exclude manuscripts without a final decision (most of which were submitted in 2021 or 2022), which may have still been in the review process at the time of data collection. This reduces the number of manuscripts to 210,868. Finally, we exclude manuscripts without both interdisciplinarity measures defined. Manuscripts not matched to OpenAlex will not have either of these measures since they rely on OpenAlex concept tags. Even for manuscripts found on OpenAlex, undefined measures could still result from absent Level 0 concept tags on the manuscript itself, absent references, or absent Level 0 concept tags on references. This reduces the number of manuscripts to 128,950. For regression models including author team covariates, the number of manuscripts reduces to 128,939 due to missing authorship data in corresponding OpenAlex records.

*SI Appendix*, Figs. S1–S4 display the distribution of final decision, submission year, number of authors, and lead author country by sample inclusion. Notably, rejected papers make up 60.5% of all submissions but only 45.1% of papers in our analytic sample, while accepted papers make up 33.0% of all submissions but 53.5% of the analytic sample. This is due to the fact that accepted submissions are published by IOPP and are thus more likely to be indexed by OpenAlex, whereas rejected submissions are only indexed if they are published elsewhere or uploaded to a preprint server such as arXiv (which is widely adopted in the physical science community). The analytic sample also overrepresents manuscripts submitted earlier in our time period of study, with Western-country lead authors, and larger team sizes (*SI Appendix*, section 2 for details).

### Regression Models.

We use logistic regression to model the relationship between each type of interdisciplinarity and two binary evaluation outcomes: final decision and reviewer recommendation to accept (henceforth “review positivity”). Our full model is

[3] $$\begin{array}{cc} \log\frac{p_{i}}{1-p_{i}} & =\alpha +\beta_{1}\left({\text{TopicID}}_{i}\right)+\beta_{2}\left({\text{Knowledge-baseID}}_{i}\right)\\ & \;+\;\beta_{3}\left({\text{TopicID}}_{i}\times \;{\text{Knowledge-baseID}}_{i}\right)\\ & \;+\;\vec{\gamma_{0}}\vec{u_{i}}\\ & \;+\;\gamma_{1}\left({\text{Journal}}_{i}\right)+\gamma_{2}\left(\text{Submission}\;{\text{year}}_{i}\right)\\ & \;+\;\epsilon_{i}, \end{array}$$

where $p_{i}$ is the probability of a positive manuscript decision (the manuscript is accepted, final decision = 1; or a reviewer recommends acceptance/revision rather than rejection, review positivity = 1). $ID_{i}$ is the normalized value of the interdisciplinarity measure (topic or knowledge base). ${\vec{u}}_{i}$ is a vector of covariates for manuscript i, including manuscript type (e.g. article, letter), number of authors (67), maximum number of prior publications and citations on the authorship team, and number of references. Numeric controls are deciled to improve model AIC, but results hold with raw numeric variables (*SI Appendix*, section 9). We also control for submission year and journal.

## Supplementary Material

Appendix 01 (PDF)

## Data Availability

Anonymized CSV files with anonymized data have been deposited in Github (68) Some study data are available. We will not be able to share the raw data, but we will share anonymized data (with a small amount of noise added to further ensure privacy).

## References

[1] National Academy of Sciences, National Academy of Engineering, Institute of Medicine, Facilitating Interdisciplinary Research (National Academies Press, Washington, D.C., 2004).

[2] P. E. Smaldino, C. O’Connor, Interdisciplinarity can aid the spread of better methods between scientific communities. Collect. Intell. **1**, 263391372211318 (2022).

[3] R. Rylance, Grant giving: Global funders to focus on interdisciplinarity. Nature **525**, 313–315 (2015). 26381969 10.1038/525313a

[4] E. Niiler, \$150M for Stanford Bio-X center. Nat. Biotechnol. **17**, 1148–1148 (1999). 10.1038/70662 10585684

[5] L. Pray, Interdisciplinarity in science and engineering: Academia in transition. Science (2002). https://www.science.org/content/article/interdisciplinarity-science-and-engineering-academia-transition. Accessed 7 March 2023.

[6] C. M. Sá, ‘Interdisciplinary strategies’ in U.S. research universities. High. Educ. **55**, 537–552 (2008).

[7] D. Rhoten, A. Parker, Risks and rewards of an interdisciplinary research path. Science **306**, 2046–2046 (2004). 15604393 10.1126/science.1103628

[8] A. Paytan, M. Lou Zoback, Crossing boundaries, hitting barriers. Nature **445**, 950–950 (2007).

[9] B. Nelson, Interdisciplinary studies: Seeking the right toolkit. Nature **476**, 115–117 (2011). 21818863 10.1038/nj7358-115a

[10] E. V. Fischer , Is Pretenure Interdisciplinary Research a Career Risk? Eos (2012), *(http://eos.org/opinions/is-pretenure-interdisciplinary-research-a-career-risk)*.

[11] M. Teplitskiy, H. Peng, A. Blasco, K. R. Lakhani, Is novel research worth doing? Evidence from peer review at 49 journals. Proc. Natl. Acad. Sci. U.S.A. **119**, e2118046119 (2022). 36395142 10.1073/pnas.2118046119 PMC9704701

[12] E. Leahey, C. M. Beckman, T. L. Stanko, Prominent but less productive: The impact of interdisciplinarity on scientists’ research. Adm. Sci. Q. **62**, 105–139 (2017).

[13] G. Hsu, M. T. Hannan, Ö. Koçak, Multiple category memberships in markets: An integrative theory and two empirical tests. Am. Sociol. Rev. **74**, 150–169 (2009).

[14] E. W. Zuckerman, T. Y. Kim, K. Ukanwa, J. von Rittmann, Robust identities or nonentities? Typecasting in the feature-film labor market Am. J. Sociol. **108**, 1018–1073 (2003).

[15] M. Leung, A. Sharkey, Out of sight, out of mind? Evidence of perceptual factors in the multiple-category discount Organ. Sci. **25**, 171–184 (2014).

[16] G. Negro, M. D. Leung, “Actual’’ and perceptual effects of category spanning. Organ. Sci. **24**, 684–696 (2013).

[17] R. Fini, J. Jourdan, M. Perkmann, L. Toschi, A, New take on the categorical imperative: Gatekeeping, boundary maintenance, and evaluation penalties in science. Organ. Sci. **34**, 1090–1110 (2022).

[18] M. Hewstone, M. Rubin, H. Willis, Intergroup bias. Annu. Rev. Psychol. **53**, 575–604 (2002). 11752497 10.1146/annurev.psych.53.100901.135109

[19] M. T. Hannan, Partiality of memberships in categories and audiences. Ann. Rev. Sociol. **36**, 159–181 (2010).

[20] B. K. Stroube, K. Vakili, M. A. Bikard, The misfit bias. Acad. Manag. Proc. **2024**, 10170 (2024).

[21] K. Huutoniemi, I. Rafols, “Interdisciplinarity in research evaluation” in The Oxford Handbook of Interdisciplinarity, R. Frodeman, Ed. (Oxford University Press, ed. 2, 2017), pp. 498–512.

[22] E. Leahey, The Perks and Perils of interdisciplinary research. Eur. Rev. **26**, S55–S67 (2018).

[23] D. van Knippenberg, Team innovation. Annu. Rev. Organ. Psych. Organ. Behav. **4**, 211–233 (2017).

[24] A. Somech, The effects of leadership style and team process on performance and innovation in functionally heterogeneous teams. J. Manag. **32**, 132–157 (2006).

[25] A. S. Alexiev, J. J. P. Jansen, F. A. J. Van den Bosch, H. W. Volberda, Top management team advice seeking and exploratory innovation: The moderating role of TMT heterogeneity. J. Manag. Stud. **47**, 1343–1364 (2010).

[26] J. G. March, Exploration and exploitation in organizational learning. Organ. Sci. **2**, 71–87 (1991).

[27] D. A. Nadler, M. L. Tushman, A model for diagnosing organizational behavior. Organ. Dyn. **9**, 35–51 (1980).

[28] K. Sombultawee, S. Boon-itt, Marketing-operations alignment: A review of the literature and theoretical background. Oper. Res. Perspect. **5**, 1–12 (2018).

[29] J. P. Cornelissen, M. D. Werner, Putting framing in perspective: A review of framing and frame analysis across the management and organizational literature. Acad. Manag. Ann. **8**, 181–235 (2014).

[30] G. Fisher, D. F. Kuratko, J. M. Bloodgood, J. S. Hornsby, Legitimate to whom? The challenge of audience diversity and new venture legitimacy J. Bus. Ventur. **32**, 52–71 (2017).

[31] S. Kaplan, Framing contests: Strategy making under uncertainty. Organ. Sci. **19**, 729–752 (2008).

[32] S. Giorgi, K. Weber, Marks of distinction: Framing and audience appreciation in the context of investment advice. Adm. Sci. Q. **60**, 333–367 (2015).

[33] E. Berkes, M. Marion, S. Milojević, B. A. Weinberg, Slow convergence: Career impediments to interdisciplinary biomedical research. Proc. Natl. Acad. Sci. U.S.A. **121**, e2402646121 (2024). 39074264 10.1073/pnas.2402646121 PMC11317606

[34] L. Bromham, R. Dinnage, X. Hua, Interdisciplinary research has consistently lower funding success. Nature **534**, 684–687 (2016). 27357795 10.1038/nature18315

[35] M. Seeber, J. Vlegels, M. Cattaneo, Conditions that do or do not disadvantage interdisciplinary research proposals in project evaluation. J. Am. Soc. Inf. Sci. Technol. **73**, 1106–1126 (2022).

[36] A. Yegros-Yegros, I. Rafols, P. D’Este, Does interdisciplinary research lead to higher citation impact? The different effect of proximal and distal interdisciplinarity PLoS One **10**, e0135095 (2015). 26266805 10.1371/journal.pone.0135095 PMC4534379

[37] J. Wang, B. Thijs, W. Glänzel, Interdisciplinarity and impact: Distinct effects of variety, balance, and disparity. PLoS One **10**, e0127298 (2015). 26001108 10.1371/journal.pone.0127298 PMC4441438

[38] X. Shi, L. A. Adamic, B. L. Tseng, G. S. Clarkson, The impact of boundary spanning scholarly publications and patents. PLoS One **4**, e6547 (2009). 19688087 10.1371/journal.pone.0006547 PMC2722725

[39] G. Abramo, C. A. D’Angelo, F. Di Costa, Do interdisciplinary research teams deliver higher gains to science? Scientometrics **111**, 317–336 (2017).

[40] B. K. AlShebli, T. Rahwan, W. L. Woon, The preeminence of ethnic diversity in scientific collaboration. Nat. Commun. **9**, 5163 (2018). 30514841 10.1038/s41467-018-07634-8 PMC6279741

[41] H. Zheng, W. Li, D. Wang, Expertise diversity of teams predicts originality and long-term impact in science and technology. SSRN [Preprint] (2022). https://www.ssrn.com/abstract=4243054 (Accessed 22 March 2023).

[42] R. Dai, L. Donohue, Q. F. Drechsler, W. Jiang, Dissemination, publication, and impact of finance research: When novelty meets conventionality*. Rev. Financ. **27**, 79–141 (2023).

[43] J. M. Levitt, M. Thelwall, Is multidisciplinary research more highly cited? A macrolevel study. J. Am. Soc. Inform. Sci. Technol. **59**, 1973–1984 (2008).

[44] M. Park, S. K. Maity, S. Wuchty, D. Wang, Interdisciplinary papers supported by disciplinary grants garner deep and broad scientific impact. arXiv [Preprint] (2023). http://arxiv.org/abs/2303.14732 (Accessed 30 March 2023). 10.1093/pnasnexus/pgag057 PMC12997532 41858746

[45] M. Thelwall , Do bibliometrics introduce gender, institutional or interdisciplinary biases into research evaluations? **52**, 104829 (2023).

[46] K. Gross, C. T. Bergstrom, Why ex post peer review encourages high-risk research while ex ante review discourages it. Proc. Natl. Acad. Sci. U.S.A. **118**, e2111615118 (2021). 34921115 10.1073/pnas.2111615118 PMC8713750

[47] J. P. Ferguson, G. Carnabuci, Risky recombinations: Institutional gatekeeping in the innovation process. Organ. Sci. **28**, 133–151 (2017).

[48] J. M. Montgomery, B. Nyhan, M. Torres, How conditioning on posttreatment variables can ruin your experiment and what to do about it. Am. J. Polit. Sci. **62**, 760–775 (2018).

[49] C. Fry, M. MacGarvie, Author Country of Origin and Attention on Open Science Platforms: Evidence from COVID-19 Preprints. Management Science **70**, 5426–5444 (2024).

[50] H. Peng, K. Lakhani, M. Teplitskiy, Acceptance in top journals shows large disparities across name-inferred ethnicities. OSF. https://osf.io/mjbxg. Accessed 6 May 2024.

[51] V. Sekara , The chaperone effect in scientific publishing. Proc. Natl. Acad. Sci. U.S.A. **115**, 12603–12607 (2018). 30530676 10.1073/pnas.1800471115 PMC6294962

[52] M. Fontana, M. Iori, F. Montobbio, R. Sinatra, New and atypical combinations: An assessment of novelty and interdisciplinarity. Res. Policy **49**, 104063 (2020).

[53] B. Uzzi, S. Mukherjee, M. Stringer, B. Jones, Atypical combinations and scientific impact. Science **342**, 468–472 (2013). 24159044 10.1126/science.1240474

[54] A. L. Porter, A. S. Cohen, J. David Roessner, M. Perreault, Measuring researcher interdisciplinarity. Scientometrics **72**, 117–147 (2007).

[55] Q. Wang, J. W. Schneider, Consistency and validity of interdisciplinarity measures. Quant. Sci. Stud. **1**, 239–263 (2020).

[56] M. Teplitskiy, E. Duede, M. Menietti, K. R. Lakhani, How status of research papers affects the way they are read and cited. **51**, 104484 (2022).

[57] IOP Publishing, *Product Catalogue 2022* (IOP Publishing, Bristol, UK, 2022).

[58] J. Priem, H. Piwowar, R. Orr, OpenAlex: A fully-open index of scholarly works, authors, venues, institutions, and concepts. arXiv [Preprint] (2022). https://arxiv.org/abs/2205.01833 (Accessed 6 December 2022).

[59] C. S. Wagner , Approaches to understanding and measuring interdisciplinary scientific research (IDR): A review of the literature. J. Informet. **5**, 14–26 (2011).

[60] I. Rafols, L. Leydesdorff, A. O’Hare, P. Nightingale, A. Stirling, How journal rankings can suppress interdisciplinary research: A comparison between Innovation Studies and Business & Management. Res. Policy **41**, 1262–1282 (2012).

[61] X. Wang , Measuring interdisciplinarity of a research system: Detecting distinction between publication categories and citation categories. Scientometrics **111**, 2023–2039 (2017).

[62] L. Cassi, R. Champeimont, W. Mescheba, É. de Turckheim, Analysing institutions interdisciplinarity by extensive use of rao-stirling diversity index. PLoS One **12**, e0170296 (2017). 28114382 10.1371/journal.pone.0170296 PMC5256946

[63] A. Stirling, A general framework for analysing diversity in science, technology and society. J. R. Soc. Interface. **4**, 707–719 (2007). 17327202 10.1098/rsif.2007.0213 PMC2373389

[64] Automated concept tagging for OpenAlex, an open index of scholarly articles (2022). https://docs.google.com/document/d/1OgXSLriHO3Ekz0OYoaoP_h0sPcuvV4EqX7VgLLblKe4/edit. Accessed 14 October 2022.

[65] Z. Shen, H. Ma, K. Wang, “A web-scale system for scientific knowledge exploration” in Proceedings of ACL 2018, System Demonstrations, F. Liu, T. Solorio, Eds. (Association for Computational Linguistics, Melbourne, Australia, 2018), pp. 87–92 ed. Fei Liu and Solorio, Thamar.

[66] G. Nigel Gilbert, Referencing as persuasion. Soc. Stud. Sci. **7**, 113–122 (1977).

[67] V. Larivière, S. Haustein, K. Börner, Long-distance interdisciplinarity leads to higher scientific impact. PLoS One **10**, e0122565 (2015). 25822658 10.1371/journal.pone.0122565 PMC4379013

[68] S. Xiang, D. M. Romero, M. Teplitskiy, evaluating-idr-replication. GitHub. https://github.com/sdxiang/evaluating-idr-replication. Deposited 3 April 2025.

