# Supplementary material for: Evaluating interdisciplinary research: Disparate outcomes for topic and knowledge base
Source: Proc Natl Acad Sci U S A. 2025 Apr 18;122(16):e2409752122. doi: 10.1073/pnas.2409752122 (PMC12037057; doi:10.1073/pnas.2409752122)
Supplement: Supplementary file 1 — Appendix 01 (PDF) [file pnas.2409752122.sapp.pdf]

# SI appendix for "Evaluating interdisciplinary research: Disparate outcomes for topic and knowledge-base"

Sidney Xiang, Daniel Romero, Misha Teplitskiy

March 5, 2025

## 1 Dataset interdisciplinarity

Manuscripts included in this analysis are those submitted to one of 62 journals in the Institute of Physics Publishing portfolio. IOP Publishing categorizes its journals into one of 7 categories: Physics, Materials, Biosciences, Environmental Sciences, Astronomy and Astrophysics, Mathematics, and Interdisciplinary [45]. Table 1 summarizes the number of journals in our dataset that fall into each category– some journals fall into two or more categories (and are counted under "Multi-category" and once under each category they appear in).

### 1.1 Measure distribution

The distribution of each interdisciplinarity measure is shown in Figures 1 and 2. Manuscripts will have zero topic interdisciplinarity if they are only tagged with a single level 0 concept. The large number of zeros in the topic interdisciplinarity histogram indicates that many of our manuscripts are topically monodisciplinary.

| **Journal Discipline** | **Count** |
|---|---|
| Physics | 22 |
| Materials | 22 |
| Biosciences | 14 |
| Environmental Sciences | 10 |
| Mathematics | 5 |
| Interdisciplinary | 10 |
| Multi-category | 18 |

**Table 1:** Representation of disciplines in dataset's journals

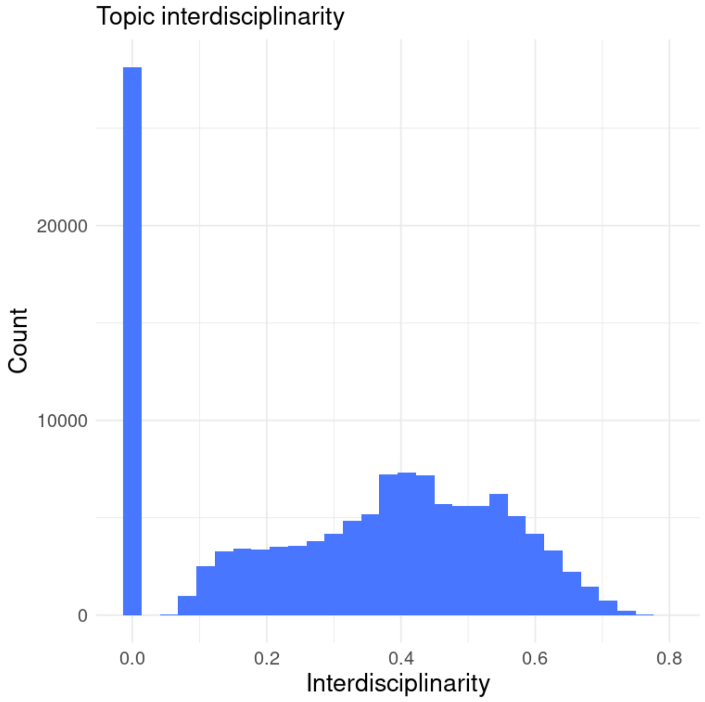


**Figure 1:** Distribution of topic interdisciplinarity in the analytic sample.

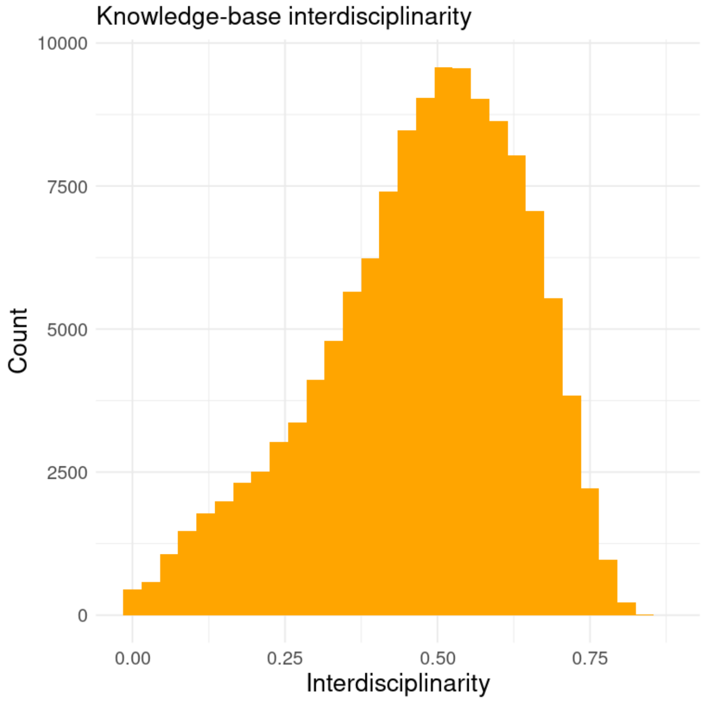


**Figure 2:** Distribution of reference interdisciplinarity in the analytic sample.

# 2 Data preprocessing

## 2.1 OpenAlex and Crossref

We supplement the IOPP administrative data with data from OpenAlex, an open-access database of academic publications, authors, venues, institutions, and concepts [1]. Manuscripts from the administrative dataset are matched to their OpenAlex records via either DOI (if available in the dataset, i.e. the submission was accepted) or fuzzy matching on the title using the fuzzywuzzy package in Python [2]. Fuzzy matching was done by querying the OpenAlex API with the manuscript title as it is listed in the administrative data, then accepting the resulting record as a match if the `fuzz.ratio` or `fuzz.partial_ratio` between the manuscript title and the record display name is greater than 90. 146,098 (66%) manuscripts are matched to OpenAlex records. Manually evaluating a random sample of matched records revealed that the incidence of mismatches was extremely low. It is important to note that all works on OpenAlex are published somewhere, whether in one of the publisher's journals, an outside journal, or on a preprint server, so we might be concerned that the matched manuscripts are mostly those accepted by the publisher. This is not the case, however; of the 146,098 matched manuscripts, 72,411 (49.6%) were accepted and 73,687 (50.4%) were rejected. Accepted manuscripts are nevertheless overrepresented in our sample, as the overall acceptance rate is 34.4%; 99.9% of accepted papers are found on OpenAlex, while only 49.9% of rejected papers are found. All OpenAlex analyses besides fuzzy matching were conducted using the May 2023 snapshot of the database.

Additionally, we used the bibliometric database *Crossref* to obtain 5 yearly random samples of size 80,000 of all works published each year for 2018 to 2022. These were used to calculate yearly similarity measures between disciplines, an important quantity for our interdisciplinarity metric.

## 2.2 Comparison of retained vs. dropped submissions

Figures 3 - 6 show the distribution of retained and dropped manuscripts over final decision (Figure 3), submission year (Figure 4), lead author country for the 10 most common countries in the data (Figure 5), and team size based on IOPP data (Figure 6). Based on these plots, we can see that rejected manuscripts and manuscripts submitted in 2021 or later are underrepresented in our analytic sample, and that manuscripts from the US, Europe, Japan, South Korea, and Russia are somewhat overrepresented compared to China, India, Iran, Pakistan, and the rest of the world. Furthermore, excluded manuscripts have a lower mean number of authors than included manuscripts (4.1 authors vs. 4.7 authors).

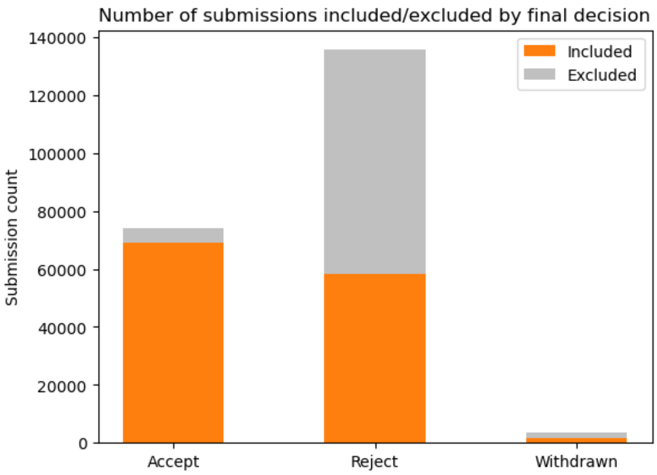


**Figure 3:** Included vs. excluded manuscripts by final decision

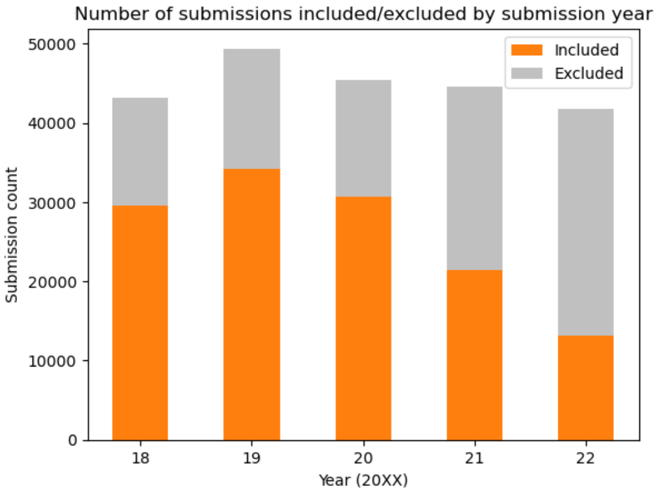


**Figure 4:** Included vs. excluded manuscripts by submission year

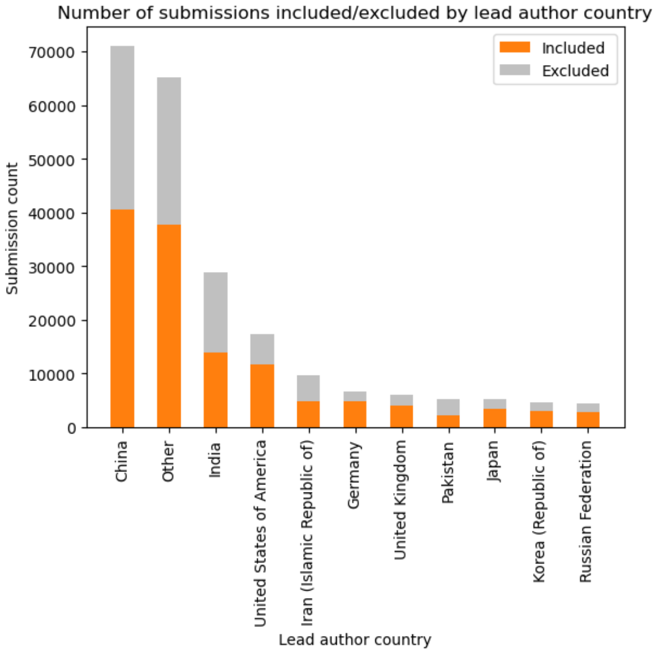


**Figure 5:** Included vs. excluded manuscripts by lead author country. Most common 10 countries in the dataset are shown, with all other countries in the "Other" category.

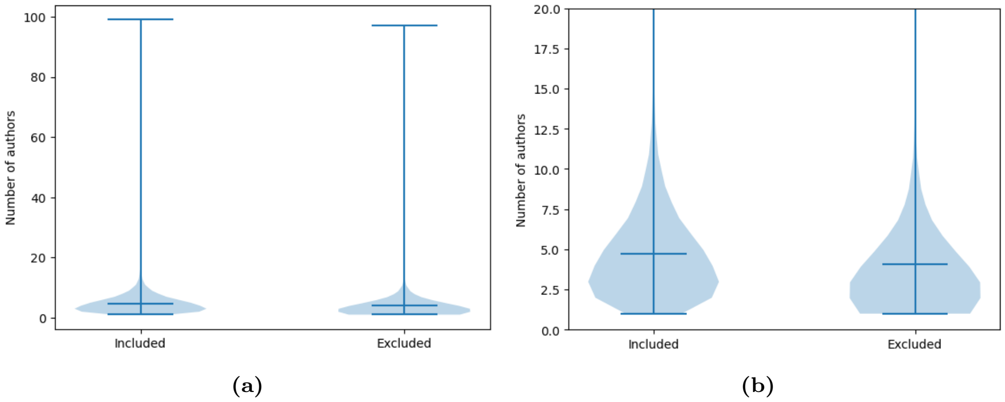


**Figure 6:** Included vs. excluded manuscripts' team size, as reported in the IOPP data. Subfigure (b) shows detail for the lower range of subfigure (a).

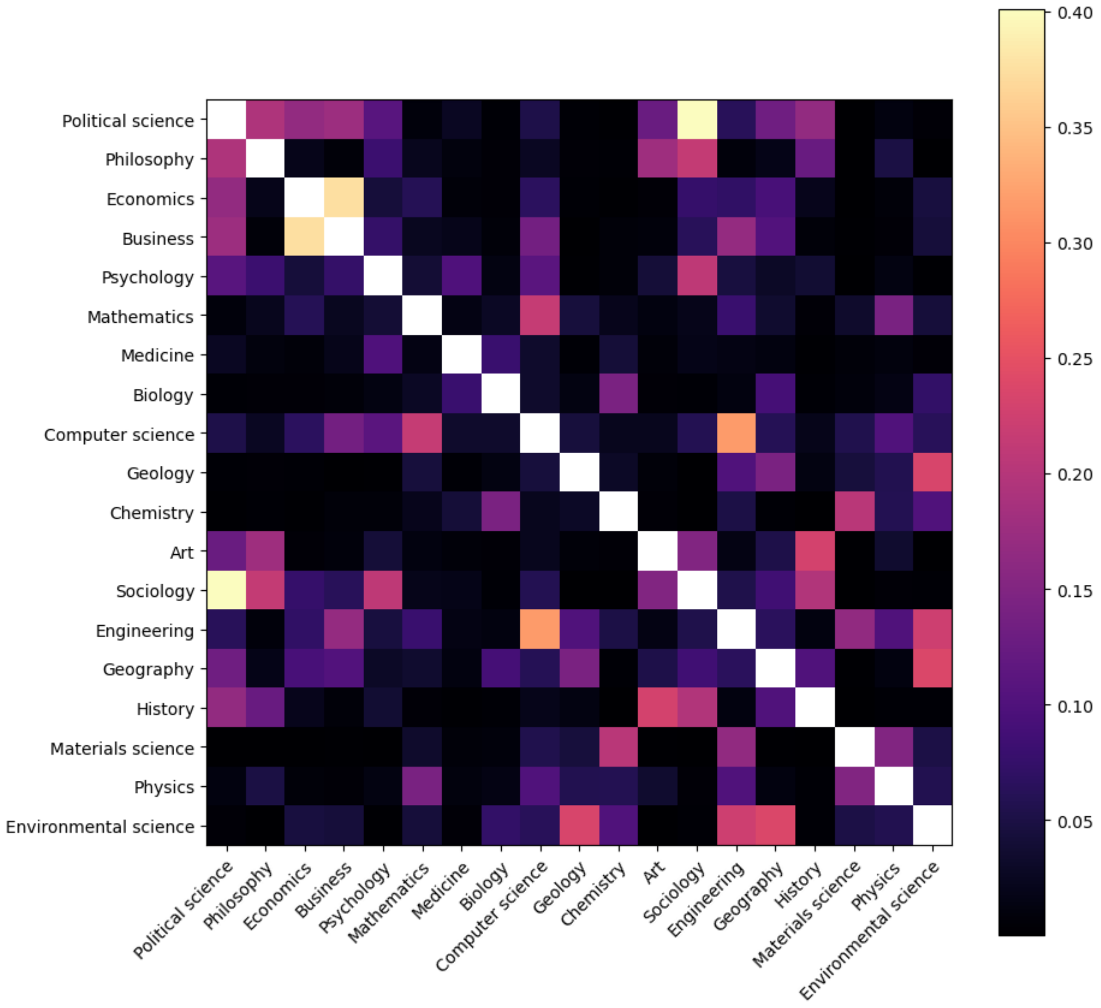


**Figure 7:** Similarity scores between Level 0 concept tags in 2018

# 3 Similarity scores

## 3.1 Similarity score visualization

Figure 7 visualizes similarity scores for level 0 concepts in 2018.

## 3.2 Sample size determination

To determine the number of samples necessary to calculate robust similarity scores for level 0 and level 1 OpenAlex concepts, we analyze the convergence of the 2018 scores as more papers are added. Results of our analysis are shown in figure 8. Similarity scores converge quickly on average as sample size increases (figures 8a, 8c), with some outliers (figures 8b, 8d) due to additional occurrences of rare concept pairs as samples are added. By 10,000 samples, the level 0 similarity scores are relatively stable, while it takes around 80,000 samples for the level 1 similarity scores to stabilize because of the larger quantity of level 1 concepts. Thus, our final similarity scores for level 0 concepts are based on a sample of 10,000 journal

articles, and our final similarity scores for level 1 concepts are based on a sample of 80,000 journal articles.

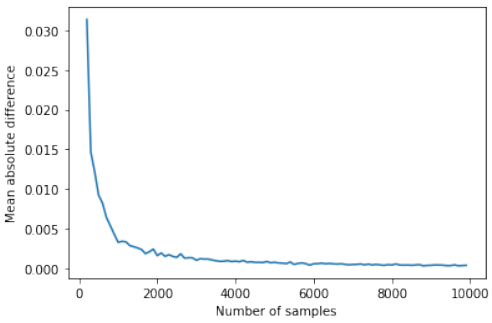


**(a)** Mean absolute difference, level 0 concepts

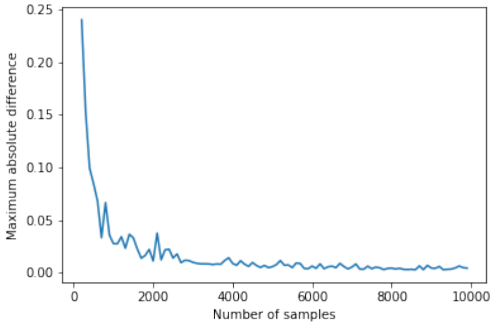


**(b)** Maximum absolute difference, level 0 concepts

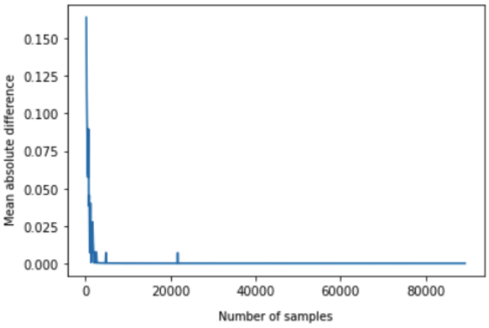


**(c)** Mean absolute difference, level 1 concepts

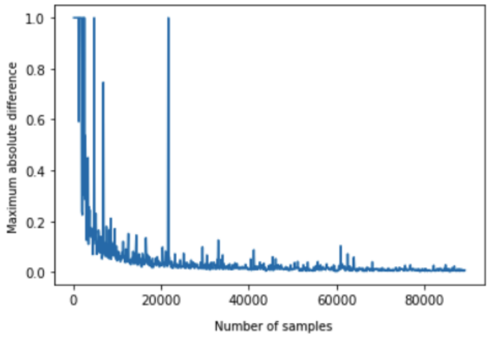


**(d)** Maximum absolute difference, level 1 concepts

**Figure 8:** To analyze similarity score convergence, similarity scores were calculated using sample sizes increasing in increments of 100. Figures 1a and 1c show the mean of the absolute difference in similarity scores between successive sample increments for level 0 and level 1 concepts, respectively. Figures 1b and 1d show the maximum of the same absolute difference for level 0 and level 1 concepts, respectively. These plots show that similarity scores converge as the sample size increases, with occasional large spikes in the maximum absolute difference for level 1 similarity scores due to first-time occurrences of new level 1 concept pairs, which peter out as sample size becomes large.

# 4 Example interdisciplinarity calculations

The integration measure of interdisciplinarity is given by the following:

$$I = 1 - \sum_{ij} s_{ij} \cdot p_i \cdot p_j \tag{1}$$

where $i$ and $j$ index disciplinary categories, $s_{ij}$ is a measure of similarity between categories $i$ and $j$, and $p_i$ and $p_j$ are the proportions of the collection made up of entities in categories $i$ and $j$.

## 4.1 Topic interdisciplinarity

Suppose a manuscript submitted in 2018 is tagged with three level 0 concept tags as follows:

| Concept tags | Score |
|---|---|
| geology | 0.6 |
| biology | 0.2 |
| chemistry | 0.1 |

Suppose the similarity between each pair of concept tags is

| Concept pair | Similarity |
|---|---|
| geology/biology | *simscore1* |
| geology/chemistry | *simscore2* |
| biology/chemistry | *simscore3* |

To calculate topic interdisciplinarity, we first sum the scores for each concept on the manuscript (0.9). Then we use the concept similarity scores and plug everything in to equation 1 to get

$$I = 1 - \left[ (simscore1 \times \frac{0.6}{0.9} \times \frac{0.2}{0.9}) + (simscore2 \times \frac{0.6}{0.9} \times \frac{0.1}{0.9}) + (simscore3 \times \frac{0.2}{0.9} \times \frac{0.1}{0.9}) \right] \tag{2}$$

## 4.2 Knowledge-base interdisciplinarity

Suppose a manuscript submitted in 2018 cites four references, *A*, *B*, *C*, and *D*. The level 0 concept tags and scores are as in table 2.

| Reference | Concept tags | Score |
|---|---|---|
| A | physics<br>materials science | 0.8<br>0.5 |
| B | physics | 0.6 |
| C | chemistry | 0.7 |
| D | materials science<br>chemistry | 0.5<br>0.2 |

**Table 2:** References for example support interdisciplinarity calculation

To calculate knowledge-base interdisciplinarity, we first sum the scores for each concept tag across references (table 3).
Using the method outlined in section 3.2, the similarity scores in 2018 between the three categories are (table 4):
Using equation 1, we calculate this manuscript's knowledge-base interdisciplinarity to be

$$\begin{aligned} I &= 1 - \left[ (0.150 \times \frac{1.4}{3.3} \times \frac{1.0}{3.3}) + (0.058 \times \frac{1.4}{3.3} \times \frac{0.9}{3.3}) + (0.204 \times \frac{1.0}{3.3} \times \frac{0.9}{3.3}) \right] \\ &= 0.957 \end{aligned} \tag{3}$$

| Concept tag | Score |
|---|---|
| physics | 1.4 |
| materials science | 1.0 |
| chemistry | 0.9 |
| **Total** | **3.3** |

**Table 3:** Total concept scores for references in table 2

| Concept pair | Similarity |
|---|---|
| physics/materials science | 0.150 |
| physics/chemistry | 0.058 |
| materials science/chemistry | 0.204 |

**Table 4:** Similarity scores between the three concept tags represented in the references in table 2

# 5 Interaction plots with review positivity as outcome variable

Figures 9 and 10 illustrate the interaction relationship between topic interdisciplinarity and knowledge-base interdisciplinarity when review positivity is used as the outcome variable. The relationship between interdisciplinarities is similar to when final decision is used as the outcome variable (figures 6 and 7 in the main text).

# 6 Regression tables for journal category analysis

Tables 5 and 6 are the regression tables for monodisciplinary and interdisciplinary journals, whose coefficients are summarized in figures 8 and 9 in the main text.

# 7 Level 1 interdisciplinarity results

As a robustness check, we remeasure both topic and knowledge-base interdisciplinarity using level 1 OpenAlex concept tags (which roughly correspond to subfield) rather than level 0 concept tags (which roughly correspond to field). Measure distributions for level 1 interdisciplinarity are shown in Figure 11, logistic regression results are shown in Tables 7 and 8, and summaries of coefficients of interest are shown in Figure 12. Notably, the effects are consistent in direction with the effects in the main text with the exception of knowledge-base interdisciplinarity and final decision.

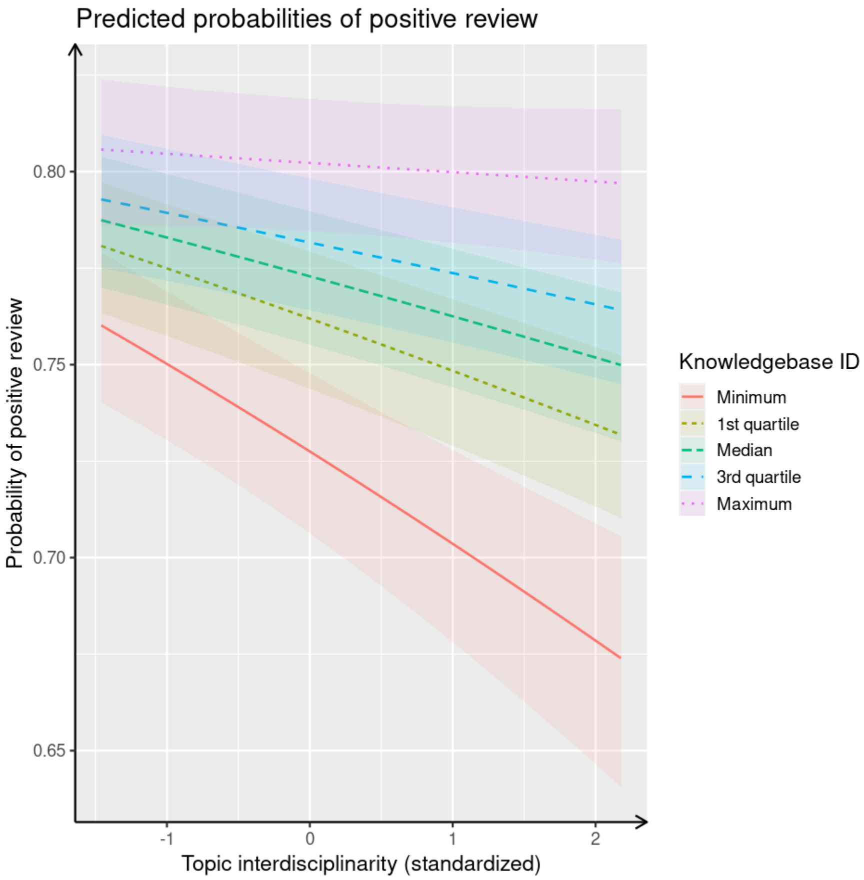


**Figure 9:** Interaction plot depicting differences in the relationship between topic interdisciplinarity and predicted review positivity (binary) for different values of knowledge-base interdisciplinarity.

**Table 5:** Logistic regression for monodisciplinary journals: Final decision

| | *Dependent variable: Final decision* | | | |
|---|---|---|---|---|
| | (1) | (2) | (3) | (4) |
| Constant | $-3.204^{***}$ | $-3.236^{***}$ | $-3.195^{***}$ | $-3.197^{***}$ |
| | (0.084) | (0.084) | (0.084) | (0.084) |
| Topic ID | $-0.158^{***}$ | | $-0.202^{***}$ | $-0.210^{***}$ |
| | (0.010) | | (0.011) | (0.011) |
| Knowledge-base ID | | $-0.012$ | $0.100^{***}$ | $0.120^{***}$ |
| | | (0.011) | (0.012) | (0.013) |
| Topic ID:Knowledge-base ID | | | | $0.072^{***}$ |
| | | | | (0.010) |
| Covariates? | *Yes* | *Yes* | *Yes* | *Yes* |
| Observations | 66,966 | 66,966 | 66,966 | 66,966 |
| Log Likelihood | -41,064.340 | -41,201.860 | -41,031.190 | -41,002.290 |
| Akaike Inf. Crit. | 82,292.680 | 82,567.730 | 82,228.380 | 82,172.580 |
| *Note:* | | | | $^{*}$p<0.1; $^{**}$p<0.05; $^{***}$p<0.01 |

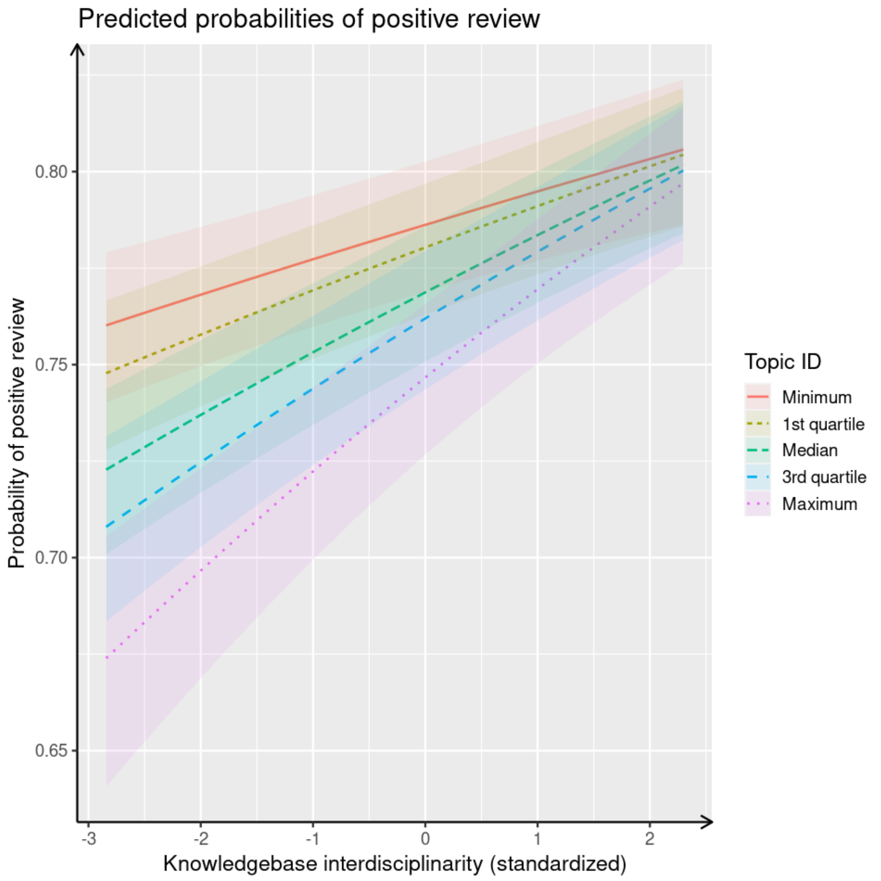


**Figure 10:** Interaction plot depicting differences in the relationship between knowledge-base interdisciplinarity and predicted review positivity (binary) for different values of topic interdisciplinarity.

**Table 6:** Logistic regression for interdisciplinary journals: Final decision

| | *Dependent variable: Final decision* | | | |
|---|---|---|---|---|
| | (1) | (2) | (3) | (4) |
| Constant | $-2.402^{***}$ | $-2.442^{***}$ | $-2.444^{***}$ | $-2.434^{***}$ |
| | (0.104) | (0.104) | (0.104) | (0.104) |
| Topic ID | $0.047^{***}$ | | 0.009 | 0.008 |
| | (0.009) | | (0.010) | (0.010) |
| Knowledge-base ID | | $0.093^{***}$ | $0.089^{***}$ | $0.084^{***}$ |
| | | (0.009) | (0.011) | (0.011) |
| Topic ID:Knowledge-base ID | | | | $-0.014^{*}$ |
| | | | | (0.009) |
| Covariates? | *Yes* | *Yes* | *Yes* | *Yes* |
| Observations | 61,973 | 61,973 | 61,973 | 61,973 |
| Log Likelihood | -38,336.570 | -38,301.780 | -38,301.430 | -38,300.070 |
| Akaike Inf. Crit. | 76,833.130 | 76,763.560 | 76,764.860 | 76,764.140 |
| *Note:* | | | | $^{*}$p<0.1; $^{**}$p<0.05; $^{***}$p<0.01 |

**Table 7:** Logistic regression: Final decision (L1)

| | *Dependent variable: Final decision* | | | | | |
|---|---|---|---|---|---|---|
| | (1) | (2) | (3) | (4) | (5) | (6) |
| Constant | 0.141*** | 0.141*** | −2.650*** | −2.652*** | −2.649*** | −2.650*** |
| | (0.006) | (0.006) | (0.071) | (0.071) | (0.071) | (0.071) |
| Topic ID | −0.048*** | | −0.035*** | | −0.036*** | −0.035*** |
| | (0.006) | | (0.006) | | (0.007) | (0.007) |
| Knowledge-base ID | | 0.025*** | | −0.011* | 0.002 | 0.013* |
| | | (0.006) | | (0.007) | (0.007) | (0.007) |
| Topic ID:Knowledge-base ID | | | | | | 0.023*** |
| | | | | | | (0.005) |
| Covariates? | *No* | *No* | *Yes* | *Yes* | *Yes* | *Yes* |
| Observations | 128,545 | 128,545 | 128,534 | 128,534 | 128,534 | 128,534 |
| Log Likelihood | -88,743.340 | -88,770.760 | -79,428.530 | -79,443.110 | -79,428.470 | -79,419.510 |
| Akaike Inf. Crit. | 177,490.700 | 177,545.500 | 159,087.100 | 159,116.200 | 159,088.900 | 159,073.000 |
| *Note:* | | | | | | *p<0.1; **p<0.05; ***p<0.01 |

**Table 8:** Logistic regression: Review positivity (L1)

| | *Dependent variable: Review positivity* | | | | | |
|---|---|---|---|---|---|---|
| | (1) | (2) | (3) | (4) | (5) | (6) |
| Constant | 1.392*** | 1.392*** | 0.619*** | 0.626*** | 0.628*** | 0.628*** |
| | (0.006) | (0.006) | (0.070) | (0.070) | (0.070) | (0.070) |
| Topic ID | −0.026*** | | −0.014** | | −0.021*** | −0.021*** |
| | (0.006) | | (0.006) | | (0.006) | (0.006) |
| Knowledge-base ID | | 0.041*** | | 0.016*** | 0.024*** | 0.030*** |
| | | (0.006) | | (0.006) | (0.007) | (0.007) |
| Topic ID:Knowledge-base ID | | | | | | 0.014*** |
| | | | | | | (0.005) |
| Covariates? | *No* | *No* | *Yes* | *Yes* | *Yes* | *Yes* |
| Observations | 196,428 | 196,428 | 196,399 | 196,399 | 196,399 | 196,399 |
| Log Likelihood | -98,061.950 | -98,046.050 | -95,251.590 | -95,250.810 | -95,245.170 | -95,241.460 |
| Akaike Inf. Crit. | 196,127.900 | 196,096.100 | 190,733.200 | 190,731.600 | 190,722.300 | 190,716.900 |

*Note:* *p<0.1; **p<0.05; ***p<0.01

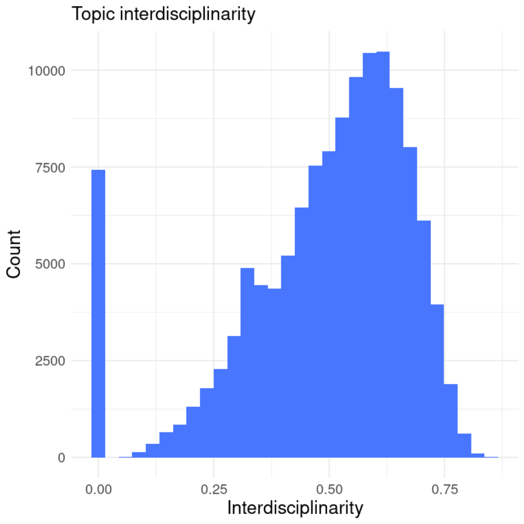


**(a)**

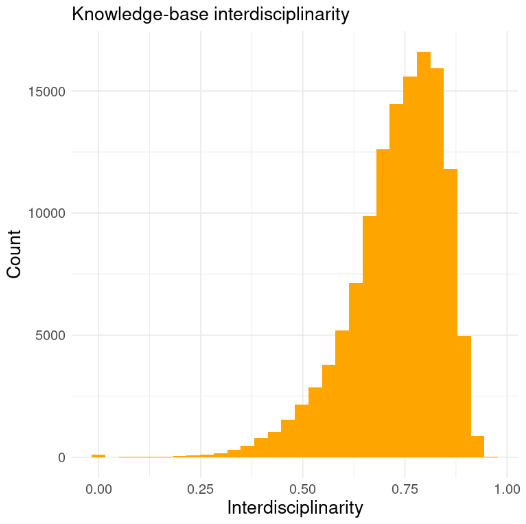


**(b)**

**Figure 11:** Measure distributions for (a) topic and (b) knowledge-base interdisciplinarity when measured using level 1 concept tags

# 8 Linear probability model results

As a robustness check, we also assess the relationship between each interdisciplinarity and evaluation outcomes using a linear probability model of the form

$$\begin{aligned} y_i = \alpha &+ \beta_1(\text{TopicID}_i) + \beta_2(\text{Knowledge-baseID}_i) \\ &+ \beta_3(\text{TopicID}_i \times \text{Knowledge-baseID}_i) \\ &+ \vec{\gamma_0}\vec{u_i} \\ &+ \gamma_1(\text{Journal}_i) + \gamma_2(\text{Submission year}_i) \\ &+ \epsilon_i \end{aligned} \tag{4}$$

Linear model results are shown in Tables 9 and 10, and coefficients of interest are summarized in Figure 13.

# 9 Results for untransformed numeric control variables

As a robustness check, we present regression results for untransformed numeric control variables. For the model in the main text, we chose to decile numeric covariates (number of authors, maximum author prior publications and citations, and number of references) because that resulted in the lowest model AIC. Tables 11 and 12 and Figure 14 show the results for untransformed numeric covariates.

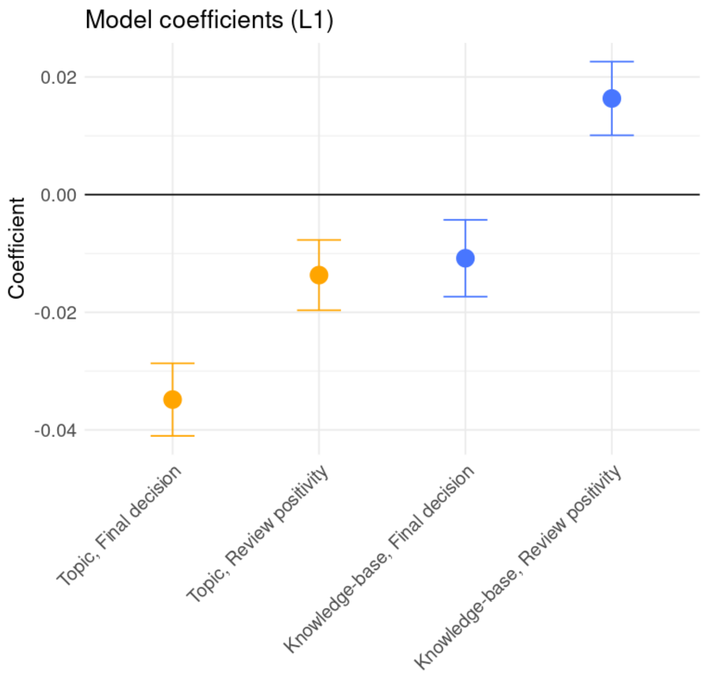


**Figure 12:** Level 1 interdisciplinarity regression coefficients and standard errors for models 3-4, which include covariates but not interactions

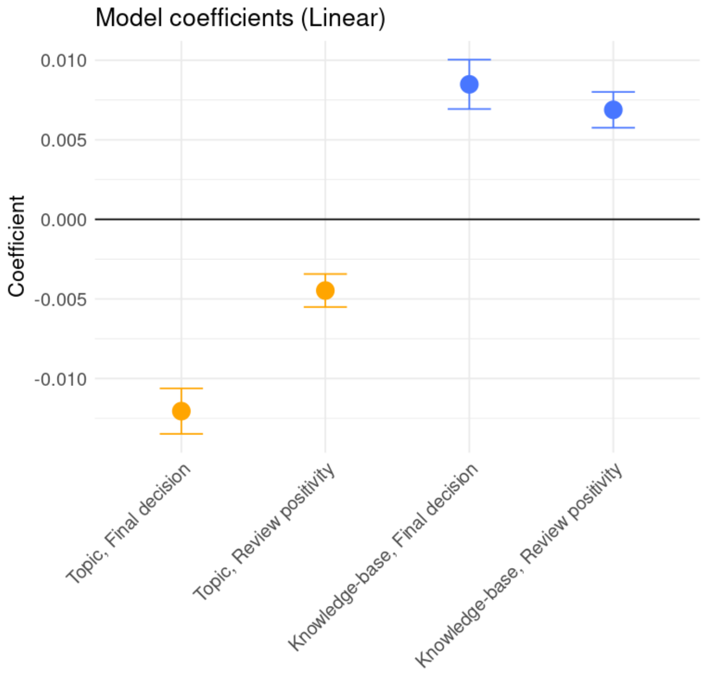


**Figure 13:** Linear model regression coefficients and standard errors for models 3-4, which include covariates but not interactions.

**Table 9:** Linear regression: Final decision

| | *Dependent variable: Final decision* | | | | | |
|---|---|---|---|---|---|---|
| | (1) | (2) | (3) | (4) | (5) | (6) |
| Constant | 0.535*** | 0.535*** | −0.064*** | −0.067*** | −0.059*** | −0.060*** |
| | (0.001) | (0.001) | (0.014) | (0.014) | (0.014) | (0.014) |
| Topic ID | −0.010*** | | −0.012*** | | −0.020*** | −0.021*** |
| | (0.001) | | (0.001) | | (0.002) | (0.002) |
| Knowledge-base ID | | 0.005*** | | 0.008*** | 0.019*** | 0.021*** |
| | | (0.001) | | (0.002) | (0.002) | (0.002) |
| Topic ID:Knowledge-base ID | | | | | | 0.009*** |
| | | | | | | (0.001) |
| Covariates? | *No* | *No* | *Yes* | *Yes* | *Yes* | *Yes* |
| Observations | 128,950 | 128,950 | 128,939 | 128,939 | 128,939 | 128,939 |
| $R^2$ | 0.000 | 0.000 | 0.133 | 0.133 | 0.134 | 0.134 |
| Adjusted $R^2$ | 0.000 | 0.000 | 0.132 | 0.132 | 0.133 | 0.134 |
| *Note:* | | | | | | *p<0.1; **p<0.05; ***p<0.01 |

**Table 10:** Linear regression: Review positivity

| | *Dependent variable: Review positivity* | | | | | |
|---|---|---|---|---|---|---|
| | (1) | (2) | (3) | (4) | (5) | (6) |
| Constant | 0.801*** | 0.801*** | 0.644*** | 0.643*** | 0.647*** | 0.647*** |
| | (0.001) | (0.001) | (0.010) | (0.010) | (0.010) | (0.010) |
| Topic ID | −0.002** | | −0.004*** | | −0.009*** | −0.010*** |
| | (0.001) | | (0.001) | | (0.001) | (0.001) |
| Knowledge-base ID | | 0.008*** | | 0.007*** | 0.011*** | 0.013*** |
| | | (0.001) | | (0.001) | (0.001) | (0.001) |
| Topic ID:Knowledge-base ID | | | | | | 0.004*** |
| | | | | | | (0.001) |
| Covariates? | *No* | *No* | *Yes* | *Yes* | *Yes* | *Yes* |
| Observations | 197,119 | 197,119 | 197,090 | 197,090 | 197,090 | 197,090 |
| $R^2$ | 0.000 | 0.000 | 0.028 | 0.028 | 0.028 | 0.028 |
| Adjusted $R^2$ | 0.000 | 0.000 | 0.027 | 0.027 | 0.027 | 0.027 |

*Note:* *p<0.1; **p<0.05; ***p<0.01

**Table 11:** Logistic regression: Final decision, untransformed covariates

| | *Dependent variable: Final decision* | | | | | |
|---|---|---|---|---|---|---|
| | (1) | (2) | (3) | (4) | (5) | (6) |
| Constant | 0.142*** | 0.142*** | −2.534*** | −2.550*** | −2.513*** | −2.522*** |
| | (0.006) | (0.006) | (0.073) | (0.073) | (0.073) | (0.073) |
| Topic ID | −0.040*** | | −0.051*** | | −0.095*** | −0.097*** |
| | (0.006) | | (0.007) | | (0.007) | (0.007) |
| Knowledge-base ID | | 0.020*** | | 0.050*** | 0.099*** | 0.111*** |
| | | (0.006) | | (0.007) | (0.008) | (0.008) |
| Topic ID:Knowledge-base ID | | | | | | 0.042*** |
| | | | | | | (0.006) |
| Covariates? | *No* | *No* | *Yes* | *Yes* | *Yes* | *Yes* |
| Observations | 128,950 | 128,950 | 128,939 | 128,939 | 128,939 | 128,939 |
| Log Likelihood | -89,031.640 | -89,050.740 | -80,798.860 | -80,804.770 | -80,724.250 | -80,702.760 |
| Akaike Inf. Crit. | 178,067.300 | 178,105.500 | 161,765.700 | 161,777.500 | 161,618.500 | 161,577.500 |
| *Note:* | | | | | | *p<0.1; **p<0.05; ***p<0.01 |

**Table 12:** Logistic regression: Review positivity, untransformed covariates

| | *Dependent variable:* | | | | | |
|---|---|---|---|---|---|---|
| | Review positivity | | | | | |
| | (1) | (2) | (3) | (4) | (5) | (6) |
| Constant | 1.391*** | 1.392*** | 0.565*** | 0.556*** | 0.582*** | 0.580*** |
| | (0.006) | (0.006) | (0.071) | (0.071) | (0.071) | (0.071) |
| Topic ID | −0.012** | | −0.027*** | | −0.061*** | −0.061*** |
| | (0.006) | | (0.007) | | (0.008) | (0.008) |
| Knowledge-base ID | | 0.049*** | | 0.050*** | 0.079*** | 0.085*** |
| | | (0.006) | | (0.007) | (0.008) | (0.008) |
| Topic ID:Knowledge-base ID | | | | | | 0.021*** |
| | | | | | | (0.006) |
| Covariates? | *No* | *No* | *Yes* | *Yes* | *Yes* | *Yes* |
| Observations | 197,119 | 197,119 | 197,090 | 197,090 | 197,090 | 197,090 |
| Log Likelihood | -98,435.400 | -98,399.480 | -95,976.200 | -95,960.850 | -95,928.300 | -95,922.710 |
| Akaike Inf. Crit. | 196,874.800 | 196,803.000 | 192,120.400 | 192,089.700 | 192,026.600 | 192,017.400 |

*Note:* *p<0.1; **p<0.05; ***p<0.01

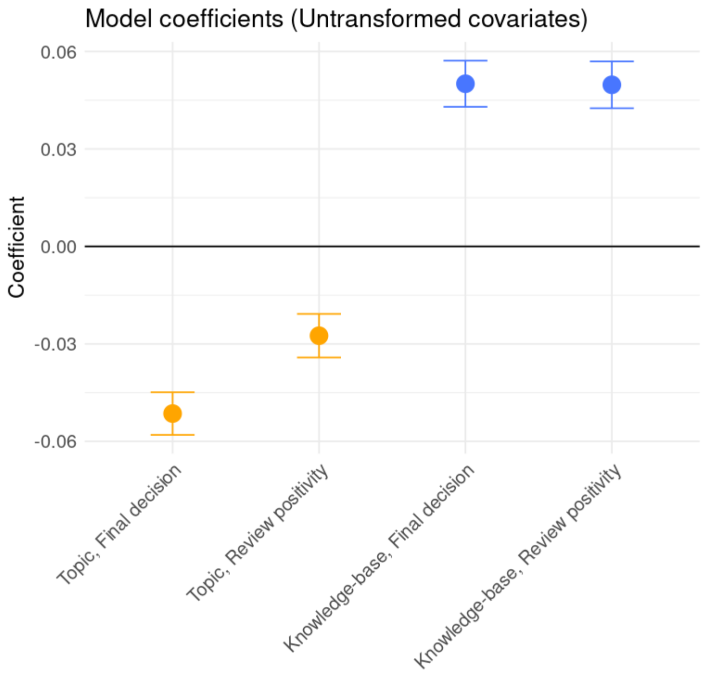


**Figure 14:** Regression coefficients for logistic model with untransformed numeric covariates for models 3-4, which include covariates but nto interactions
